# Light Exposure at Night and Cardiovascular Disease Incidence

**DOI:** 10.1001/jamanetworkopen.2025.39031

**Published:** 2025-10-23

**Authors:** Daniel P. Windred, Angus C. Burns, Martin K. Rutter, Jacqueline M. Lane, Richa Saxena, Frank A. J. L. Scheer, Sean W. Cain, Andrew J. K. Phillips

**Affiliations:** 1 Flinders Health and Medical Research Institute (Sleep Health), Flinders University, Bedford Park, South Australia, Australia; 2 Division of Sleep and Circadian Disorders, Brigham and Women’s Hospital, Boston, Massachusetts; 3 Division of Sleep Medicine, Harvard Medical School, Boston, Massachusetts; 4 Program in Medical and Population Genetics, Broad Institute, Cambridge, Massachusetts; 5 Center for Genomic Medicine, Massachusetts General Hospital, Boston; 6 Division of Diabetes, Endocrinology and Gastroenterology, School of Medical Sciences, University of Manchester, Manchester, United Kingdom; 7 Diabetes, Endocrinology and Metabolism Centre, Manchester University National Health Service Foundation Trust, Manchester Academic Health Science Centre, National Institute for Health and Care Research Manchester Biomedical Research Centre, Manchester, United Kingdom; 8 Department of Anesthesia, Critical Care and Pain Medicine, Massachusetts General Hospital, Harvard Medical School, Boston

## Abstract

**Importance:**

Light at night causes circadian disruption, which is a known risk factor for adverse cardiovascular outcomes. However, it is not well understood of cardiovascular diseases.

**Objective:**

To assess whether day and night light exposure is associated with incidence of cardiovascular diseases, and whether associations of light with cardiovascular diseases differ according to genetic susceptibility, sex, and age.

**Design, Setting, and Participants:**

This prospective cohort study analyzed cardiovascular disease records across 9.5 years (June 2013 to November 2022) from UK Biobank participants who wore light sensors in a naturalistic setting. Data were analyzed from September 2024 to July 2025.

**Exposure:**

Approximately 13 million hours of light exposure data, tracked by wrist-worn light sensors (1 week each), categorized into the 0 to 50th, 51st to 70th, 71st to 90th, and 91st to 100th percentiles.

**Main Outcomes and Measures:**

Incidence of coronary artery disease, myocardial infarction, heart failure, atrial fibrillation, and stroke after light tracking were derived from UK National Health Service records. Risks of cardiovascular diseases were assessed using Cox proportional hazards models (3 primary models adjusted at 3 levels) and reported as hazard ratios (HRs).

**Results:**

A total of 88 905 individuals were included (mean [SD] age, 62.4 [7.8] years; 50 577 female [56.9%]). Compared with individuals with dark nights (0-50th percentiles), those with the brightest nights (91st-100th percentiles) had significantly higher risks of developing coronary artery disease (adjusted HR [aHR], 1.32; 95% CI, 1.18-1.46), myocardial infarction (aHR, 1.47; 95% CI, 1.26-1.71), heart failure (aHR, 1.56; 95% CI, 1.34-1.81), atrial fibrillation (aHR, 1.32; 95% CI, 1.18-1.46), and stroke (aHR, 1.28; 95% CI, 1.06-1.55). These associations were robust after adjusting for established cardiovascular risk factors, including physical activity, smoking, alcohol, diet, sleep duration, socioeconomic status, and polygenic risk. Larger-magnitude associations of night light with risks of heart failure (*P* for interaction = .006) and coronary artery disease (*P* for interaction = .02) were observed for females, and larger-magnitude associations of night light with risks of heart failure (*P* for interaction = .04) and atrial fibrillation (*P* for interaction = .02) were observed for younger individuals in this cohort.

**Conclusions and Relevance:**

In this cohort study, night light exposure was a significant risk factor for developing cardiovascular diseases among adults older than 40 years. These findings suggest that, in addition to current preventive measures, avoiding light at night may be a useful strategy for reducing risks of cardiovascular diseases.

## Key Points

**Question:**

Is personal light exposure at night associated with cardiovascular disease incidence?

**Findings:**

In this cohort study of 88 905 adults aged older than 40 years, exposure to brighter light at night was associated with higher risks of coronary artery disease, myocardial infarction, heart failure, atrial fibrillation, and stroke, independent of established cardiovascular risk factors.

**Meaning:**

These findings suggest that avoiding exposure to night light may lower risk of cardiovascular diseases.

## Teaser

This cohort study assesses associations of day and night light exposure with incidence of coronary artery disease, myocardial infarction, heart failure, atrial fibrillation, and stroke among adults older than 40 years in the UK.

## Introduction

Robust circadian rhythms are vital for healthy cardiovascular function. Circadian rhythms have been observed in systolic and diastolic blood pressure^1,2^; platelet activation^3^; fibrinolysis^4^; vascular endothelial function^5^; circulating cortisol, epinephrine, and norepinephrine^2^; glucose tolerance^6^; and heart rate average, heart rate variability, QT interval, and PR segment.^1,7,8^ Short-term circadian disruption in humans causes hypercoagulability,^9^ elevated heart rate,^10^ elevated blood pressure, inflammation, and reduced cardiac vagal modulation.^11,12^ Long-term circadian disruption in animal models causes myocardial fibrosis, hypertrophy, impaired contractility, adverse cardiac remodeling, and accelerated progression to heart failure.^13,14,15^ Epidemiological evidence demonstrates higher risks of adverse cardiovascular events, coronary heart disease, heart failure, atrial fibrillation, and mortality due to cardiovascular disease in rotating shift workers^16,17,18,19,20^ who have long-term exposure to circadian disruption.

Light at night causes circadian disruption,^21,22,23^ and is therefore a potential risk factor for cardiovascular diseases. Higher risks for coronary artery disease^24^ and stroke^25^ have been observed in people living in urban environments with brighter outdoor night light, as measured by satellite. Brighter night light has been cross-sectionally associated with atherosclerosis,^26,27^ obesity, hypertension, and diabetes^28^ in small but well-characterized cohorts, using bedroom^26,27^ and wrist-worn^28^ light sensors. Moreover, experimental exposure to night light elevates heart rate and alters sympathovagal balance.^29^ However, current evidence associating night light with cardiovascular risk is mostly within small cohorts or relies on geospatial-level measurements of outdoor lighting, rather than measures of personal light exposure.^30,31^

Using data captured from wrist-worn light sensors in approximately 89 000 UK Biobank participants, we recently observed higher risk of mortality by cardiometabolic causes in those exposed to brighter nights and darker days.^32^ In the same cohort, brighter nights also predicted higher incidence of type 2 diabetes,^33^ an established risk factor for cardiovascular diseases.^34^ We therefore assessed whether personal day and night light exposures were associated with incident coronary artery disease, myocardial infarction, heart failure, atrial fibrillation, and stroke, over 9.5 years of follow-up in UK Biobank participants.

## Methods

### Overview

This cohort study was granted ethical approval by the North West Multicenter Research Ethics Committee and adhered to the Strengthening the Reporting of Observational Studies in Epidemiology (STROBE) reporting guideline. Approximately 502 000 UK Biobank participants were recruited between 2006 and 2010, and 103 669 participants wore light-tracking devices (Axivity AX3; peak spectral sensitivity 560 nm) on their dominant wrist for 1 week between 2013 and 2016. Incident cardiovascular diseases were recorded up to November 2022. Detailed information on the data collection protocol, including participant consent, is available on the UK Biobank website (eTable 1 in Supplement 1).

### Exposure: Personal Light Tracking

Extraction of personal day and night light exposures from wrist-worn light sensor data in this cohort has been previously reported.^32,33,35^ In short, sensor data (100 Hz) were downsampled, cleaned for periods of nonwear and data corruption, and transformed according to the device manual and subsequent testing under reference lighting conditions^35^ (eMethods in Supplement 1). Data were then grouped into 24-hour light exposure profiles for each participant, represented by mean light exposure within each of the 48 half-hour clock time intervals (eg, all light between 12:00 AM to 12:30 AM). Factor analysis was applied to the 24-hour light exposure profiles.^35^ This unsupervised analysis revealed 2 temporal light exposure clusters, which we labeled as day (07:30 AM to 8:30 PM) and night (12:30 AM to 06:00 AM) (eMethods in Supplement 1).

### Outcome: Incident Cardiovascular Diseases

**Figure 1.**
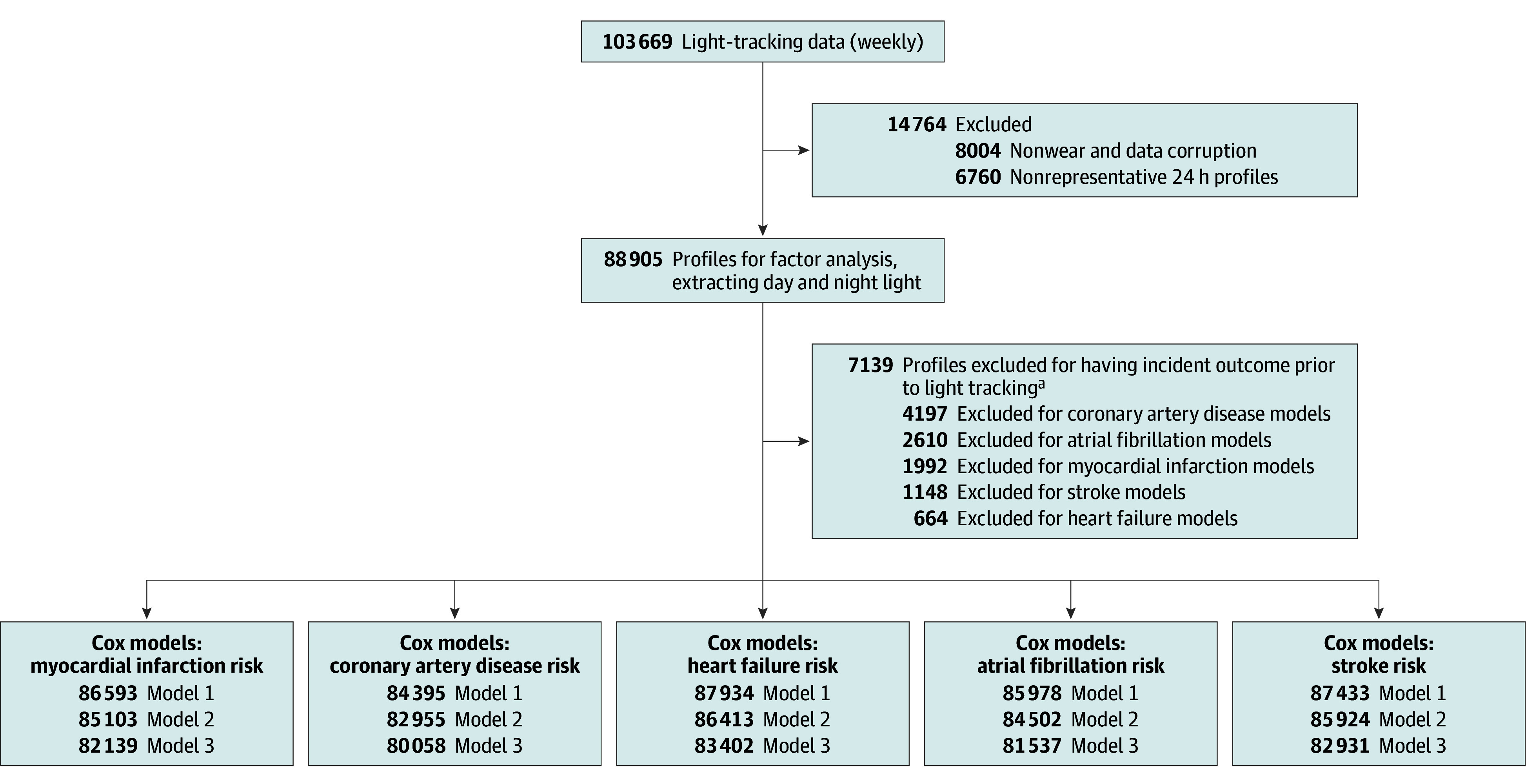
Participant Flow Diagram This figure details participant-level exclusions between data collection and final analyses for each of the 5 incident cardiovascular outcomes. ^a^Categories are not mutually exclusive.

Diagnoses of cardiovascular diseases were derived from hospital admissions, primary care, self-report, and death register records, according to *International Classification of Diseases, Ninth Revision (ICD-9)* and *International Statistical Classification of Diseases and Related Health Problems, Tenth Revision (ICD-10)* criteria (eMethods in Supplement 1). Myocardial infarction and stroke were defined according to the UK Biobank algorithmically defined outcomes. Myocardial infarction included ST-segment–elevated and non–ST-segment–elevated events. Stroke captured ischemic stroke, intracerebral hemorrhage, and subarachnoid hemorrhage. *ICD-9* and *ICD-10* codes included in myocardial infarction and stroke definitions are documented on the UK Biobank website (eTable 1 in Supplement 1). Coronary artery disease captured acute and chronic ischemic heart disease, myocardial infarction, and coronary artery operations. Atrial fibrillation was defined as the first occurrence of *ICD-10* code I48 or operation for atrial fibrillation. Heart failure was defined as the first occurrence of *ICD-10* code I50. Participants with each cardiovascular outcome prior to light tracking were excluded from analyses (Figure 1). Diagnoses across 9.5 years between June 2013 (first light tracking) and November 2022 (last follow-up) were included.

### Covariates

Participant age, sex, ethnicity, yearly household income, education level, employment status, material deprivation (Townsend Deprivation Index; scores capture areas with higher [positive score] or lower [negative score] material deprivation, relative to areas with average material deprivation [score of 0]),^36^ urbanicity of residential location, alcohol consumption (days per week), smoking status (never, previous, or current), healthy diet score,^37^ and shift work status were derived from questionnaires administered at a baseline assessment center visit (2006 to 2010). UK Biobank-defined ethnicity categories were Asian (Bangladeshi, Indian, Pakistani, or other [ie, any Asian ethnicity not otherwise specified]), Black (African, Caribbean, or other [ie, any Black ethnicity not otherwise specified]), Chinese, multiethnic (referred to as mixed in the UK Biobank; White and Asian, White and Black African, White and Black Caribbean, or other [ie, any multiethnic identity not otherwise specified]); White (British, Irish, or other [ie, any White ethnicity not otherwise specified]); and other ethnic group (ie, any ethnic group not otherwise specified); ethnicity was included as a covariate due to known differences in adverse cardiovascular outcomes between people who identify as White ethnicity and other ethnic groups. Photoperiod was defined as the time between sunrise and sunset during light tracking, capturing seasonality. Average physical activity was estimated from weekly accelerometer recordings.^38^ Preexisting diabetes was defined as occurrence of *ICD-10* codes E10 or E11, or self-reported diagnosis, prior to light tracking. Preexisting hypertension was defined as the occurrence of *ICD-10* codes I10 to I13 or I15, or measured hypertension at baseline physical assessment, prior to light tracking. Body mass index (calculated as weight in kilograms divided by height in meters squared) and cholesterol ratio were derived from physical measurements collected during baseline assessment. Sleep duration and sleep efficiency were estimated from weekly accelerometer recordings using a validated sleep-wake estimation method (GGIR package in R version 4.0.0 [R Project for Statistical Computing]), as reported previously.^39,40,41^ Polygenic risk scores for coronary artery disease,^42^ myocardial infarction,^42^ heart failure,^43^ atrial fibrillation,^42^ and stroke^44^ were generated using polygenic risk score–continuous shrinkage,^45^ and scored in the UK Biobank actigraphy cohort using PLINK 2 (Human Longevity Inc).^46^ Detailed covariate descriptions are provided in eTable 2 in Supplement 1.

### Statistical Analysis

Risks of cardiovascular diseases were assessed using Cox proportional hazards models, including day and night light as exposures (survival package in R 4.5.0; 2-sided statistical tests) (eMethods in Supplement 1). Day and night light were split into 0 to 50th, 51st to 70th, 71st to 90th, and 91st to 100th light exposure percentiles in these models. The 0 to 50th percentile reference groups captured participants in the darkest environments. Time since light tracking was used as the timescale in all models. Data were right-censored at the end of the observation period (November 29, 2022), or at participant mortality if this occurred earlier.

Primary models were adjusted at 3 levels. Model 1 adjusted for age, sex, ethnicity, and photoperiod. Model 2 additionally adjusted for education, employment, income, and deprivation. Model 3 further adjusted for physical activity, smoking status, alcohol consumption, diet, and urbanicity. Supplementary models included separate adjustments of model 3 for preexisting diabetes, hypertension, high body mass index, high cholesterol ratio, short sleep, long sleep, sleep efficiency, exclusion of shift workers, and lifestyle adjustments excluding physical activity. Selected covariates were potential confounders of associations of light exposure with cardiovascular risks. Covariates in model 3 and supplementary models were also potential mediators of these associations.

Interactions of night light exposure with age (continuous), sex, and polygenic risk were assessed in additional Cox models predicting cardiovascular outcomes (eMethods in Supplement 1). First, dose-response associations of night light with risk of each cardiovascular outcome were assessed by including night light as a log-linear exposure in model 3. Subsequently, interactions of age and sex with log-linear night light were added to model 3. Additionally, an interaction of polygenic risk score with log-linear night light was added to model 3. Models including polygenic risk scores were restricted to individuals of European ancestry and adjusted for the top 5 principal components of ancestry, to control for potential residual population stratification within the European ancestry subpopulation (eMethods in Supplement 1). Interaction of night light exposure with chronotype was included as a supplementary analysis. Analyses were conducted from September 2024 to July 2025 using R 4.5.0. A 2-sided P < .05 was considered significant.

## Results

The 88 905 participants included in the analyses (mean [SD] age, 62.4 [7.8] years; 50 577 female [56.9%]; 805 Asian [0.9%]; 711 Black [0.8%]; 85 924 White [97.0%]) had light data across all clock times and were free of each cardiovascular outcome at the time of light tracking (Figure 1). Mean (SD) time between light tracking and the final follow-up (November 2022 or participant mortality) was 7.9 [1.0] years. Participant characteristics and cardiovascular disease case numbers, split by light exposure percentiles, are provided in Table 1 and eTables 3 to 5 in Supplement 1.

**Table 1.** Participant Characteristics and Cardiovascular Disease Cases by Day and Night Light Exposure Percentile Groups

Characteristic	Participants, No. (%) (N = 88 905)							
	Night light exposure percentile				Day light exposure percentile			
	0-50 (n = 44 453)	51-70 (n = 17 780)	71-90 (n = 17 781)	91-100 (n = 8891)	0-50 (n = 44 453)	51-70 (n = 17 780)	71-90 (n = 17 781)	91-100 (n = 8891)
Age, mean (SD) [range], y	62.8 (7.9) [43.5 to 78.9]	61.8 (7.9) [43.5 to 79.0]	62.0 (7.8) [43.7 to 78.8]	62.4 (7.7) [43.8 to 78.4]	62.1 (8.0) [43.6 to 79.0]	62.4 (7.8) [43.6 to 78.7]	62.6 (7.7) [43.5 to 78.8]	63.5 (7.4) [43.5 to 78.5]
Sex								
Male	18 353 (41.3)	7998 (45.0)	7949 (44.7)	4022 (45.2)	18 774 (42.2)	7478 (42.1)	7719 (43.4)	4351 (48.9)
Female	26 097 (58.7)	9781 (55.0)	9831 (55.3)	4868 (54.8)	25 674 (57.8)	10 301 (57.9)	10 062 (56.6)	4540 (51.1)
Ethnicity^a^								
Asian	329 (0.7)	179 (1.0)	192 (1.1)	105 (1.2)	495 (1.1)	153 (0.9)	131 (0.7)	26 (0.3)
Black	217 (0.5)	148 (0.8)	216 (1.2)	130 (1.5)	466 (1.1)	140 (0.8)	80 (0.5)	25 (0.3)
Chinese	88 (0.2)	34 (0.2)	45 (0.3)	27 (0.3)	119 (0.3)	39 (0.2)	29 (0.2)	7 (0.1)
Multiethnic (mixed)	200 (0.5)	113 (0.6)	112 (0.6)	68 (0.8)	268 (0.6)	110 (0.6)	84 (0.5)	31 (0.3)
White	43 296 (97.4)	17 133 (96.4)	17 033 (95.8)	8462 (95.2)	42 669 (96.0)	17 190 (96.7)	17 315 (97.4)	8750 (98.4)
Other	187 (0.4)	107 (0.6)	116 (0.7)	63 (0.7)	274 (0.6)	94 (0.5)	77 (0.4)	28 (0.3)
Employed	26 360 (59.7)	11 388 (64.5)	11 474 (65.0)	5622 (63.7)	28 201 (63.9)	10 903 (61.8)	10 720 (60.7)	5020 (56.9)
Annual income, £								
<18 000	5679 (12.9)	2307 (13.1)	2278 (12.9)	1238 (14.0)	5959 (13.5)	2283 (12.9)	2221 (12.6)	1039 (11.7)
18 000-29 999	9753 (22.1)	3779 (21.4)	3747 (21.2)	1924 (21.8)	9537 (21.6)	3884 (22.0)	3815 (21.6)	1967 (22.2)
30 000-51 999	11 452 (25.9)	4637 (26.3)	4592 (26.0)	2231 (25.3)	11 431 (25.9)	4489 (25.4)	4662 (26.4)	2330 (26.3)
52 000-100 000	10 016 (22.7)	4073 (23.1)	4129 (23.4)	1957 (22.2)	10 058 (22.8)	4017 (22.8)	4079 (23.1)	2021 (22.8)
>100 000	2767 (6.3)	1219 (6.9)	1261 (7.1)	644 (7.3)	2917 (6.6)	1147 (6.5)	1220 (6.9)	607 (6.9)
Education								
Other	21 144 (48.1)	8529 (48.4)	8547 (48.6)	4300 (48.9)	21 013 (47.8)	8552 (48.7)	8577 (48.7)	4378 (49.6)
University	19 105 (43.4)	7639 (43.4)	7657 (43.5)	3775 (42.9)	19 367 (44.0)	7596 (43.2)	7566 (43.0)	3647 (41.4)
Townsend Deprivation Index, mean (SD) [range]	−1.92 (2.70) [−6.26 to 10.50]	−1.69 (2.82) [−6.26 to 9.89]	−1.61 (2.88) [−6.26 to 9.99]	−1.39 (2.99) [−6.26 to 9.89]	−1.58 (2.90) [−6.26 to 10.50]	−1.78 (2.76) [−6.26 to 9.89]	−1.92 (2.69) [−6.26 to 9.89]	−2.25 (2.44) [−6.26 to 8.94]
Smoking								
Previous	15 409 (34.8)	6391 (36.1)	6725 (37.9)	3439 (38.8)	15 579 (35.2)	6396 (36.1)	6539 (36.9)	3450 (38.9)
Current	2411 (5.4)	1288 (7.3)	1451 (8.2)	907 (10.2)	3130 (7.1)	1247 (7.0)	1167 (6.6)	513 (5.8)
Alcohol, d/wk, mean (SD)	2.97 (2.48)	2.98 (2.50)	2.99 (2.51)	2.94 (2.55)	2.87 (2.48)	2.98 (2.50)	3.07 (2.51)	3.29 (2.53)
Urbanicity >10 000 population	36 620 (83.2)	14 741 (83.7)	15 075 (85.6)	7598 (86.5)	37 603 (85.5)	14 799 (84.1)	14 628 (83.1)	7004 (79.4)
Physical activity, mean (SD) [range], m^2^/s	27.9 (7.9) [4.8 to 69.2]	28.6 (8.3) [6.5 to 69.3]	28.5 (8.2) [5.9 to 69.2]	27.8 (8.3) [5.1 to 69.4]	27.3 (7.9) [5.1 to 69.3]	28.2 (8.0) [4.9 to 67.9]	29.0 (8.1) [6.5 to 69.4]	30.6 (8.5) [4.8 to 67.4]
Diet score: healthy	11 476 (26.6)	4362 (25.2)	4298 (24.9)	2123 (24.7)	10 920 (25.3)	4468 (25.8)	4530 (26.2)	2341 (26.9)
Photoperiod, mean (SD), h	12.2 (3.0)	12.9 (3.2)	12.8 (3.3)	12.6 (3.4)	10.9 (2.9)	12.9 (2.9)	14.5 (2.3)	15.7 (1.5)
Cardiovascular cases								
Coronary artery disease	2817 (6.5)	1202 (6.9)	1265 (7.3)	678 (7.8)	2921 (6.7)	1186 (6.8)	1204 (6.9)	651 (7.5)
Myocardial infarction	773 (1.8)	358 (2.1)	379 (2.2)	219 (2.5)	838 (1.9)	334 (1.9)	348 (2.0)	209 (2.4)
Heart failure	917 (2.1)	400 (2.3)	395 (2.8)	247 (2.9)	989 (2.3)	406 (2.3)	364 (2.1)	200 (2.3)
Atrial fibrillation	2805 (6.5)	1138 (6.6)	1204 (6.9)	683 (7.9)	2839 (6.5)	1189 (6.8)	1132 (6.5)	670 (7.7)
Stroke	1058 (2.4)	425 (2.5)	440 (2.5)	241 (2.8)	1110 (2.6)	424 (2.4)	430 (2.5)	200 (2.3)
Light exposure, median (IQR) [range], lux	0.62 (0.49 to 0.80) [0 to 1.21]	2.48 (1.64 to 3.93) [1.21 to 6.28]	16.37 (10.11 to 27.21) [6.28 to 48.30]	105.30 (69.39 to 191.13) [48.31 to 6404.29]	426.48 (221.23 to 675.17) [4.29 to 991.19]	1322.06 (1146.79 to 1520.91) [991.19 to 1747.47]	2311.83 (2004.62 to 2680.61) [1747.49 to 3140.92]	3814.79 (3446.61 to 4345.31) [3141.08 to 7887.64]

^a^ UK Biobank-defined ethnicity categories are listed in the Methods section.

### Night and Day Light Exposure Associates With Cardiovascular Disease Risk

**Figure 2.**
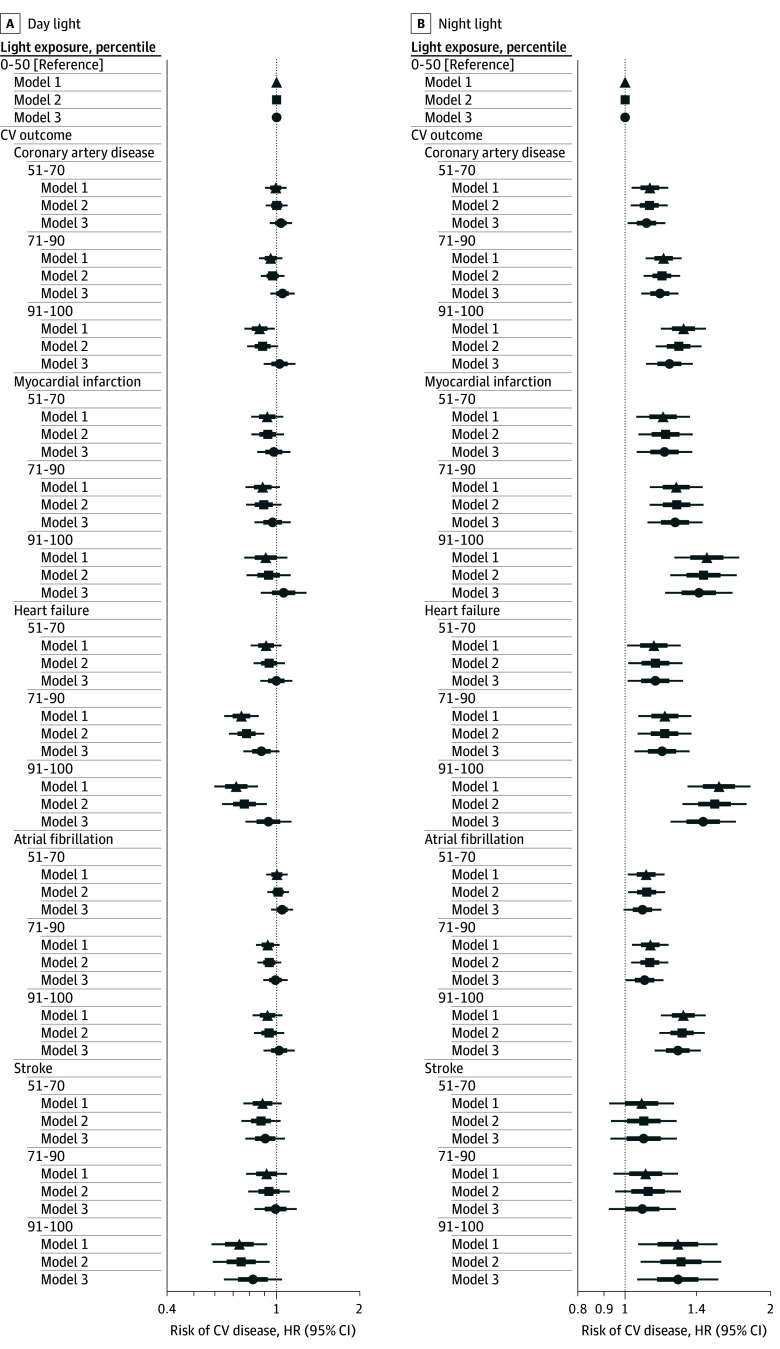
Risk of Cardiovascular Diseases Within Day and Night Light Exposure Percentile Groups Hazard ratios (HRs) and 95% CIs are adjusted for age, sex, race and ethnicity, and photoperiod (model 1); additionally adjusted for education, employment, income, and deprivation (model 2); and further adjusted for physical activity, smoking status, alcohol consumption, diet, and urbanicity (model 3). Participants with the darkest environments (0-50th percentiles) were the referent group for all models. HRs (95% CIs) are presented numerically in Table 2.

Exposure to night light was associated with higher cardiovascular disease risks, with dose-dependent associations observed (Figure 2, Table 2, and eTable 4 in Supplement 1). Compared with people with dark nights (0 to 50th light exposure percentiles), there was higher risk of coronary artery disease in individuals with brighter nights in the 51st to 70th percentiles of light exposure (model 1: hazard ratio [HR], 1.12; 95% CI, 1.03-1.23), 71st to 90th percentiles of light exposure (model 1: HR, 1.20; 95% CI, 1.10-1.31), and 91st to 100th percentiles of light exposure (model 1: 1.32; 95% CI, 1.18-1.46). Similarly, those with brighter nights had higher risk of myocardial infarction across the 51st to 70th percentiles (model 1: HR, 1.20; 95% CI, 1.05-1.36), 71st to 90th percentiles (model 1: HR, 1.27; 95% CI, 1.12-1.44), and 91st to 100th percentiles (model 1: HR, 1.47; 95 CI, 1.26-1.71), as well as a higher risk of heart failure across the 51st to 70th percentiles (model 1: HR, 1.15; 95% CI, 1.01-1.30), 71st to 90th percentiles (model 1: HR, 1.21; 95% CI, 1.06-1.37) , and 91st to 100th percentiles (model 1: HR, 1.56; 95% CI, 1.34-1.81). Those with the brightest nights (91st-100th percentiles) had higher risk for atrial fibrillation (model 1: HR, 1.32; 95% CI, 1.18-1.46), and stroke (model 1: HR, 1.28; 95% CI, 1.06-1.55) compared with those with dark nights (0-50th percentiles). Significant log-linear associations of brighter night light with higher cardiovascular disease risks were observed for all outcomes (Table 3). Associations of night light with cardiovascular outcomes were robust following adjustment for socioeconomic and lifestyle factors across models 2 and 3 (Figure 2 and Table 2).

**Table 2.** Risk of Cardiovascular Outcomes According to Light Exposure Percentile Groups Across Models 1 to 3^a^

Light exposure by percentile	Coronary artery disease, HR (95% CI)	*P* value	Myocardial infarction, HR (95% CI)	*P* value	Heart failure, HR (95% CI)	*P* value	Atrial fibrillation, HR (95% CI)	*P* value	Stroke, HR (95% CI)	*P* value
Model 1										
Night										
0-50	1 [Reference]	NA	1 [Reference]	NA	1 [Reference]	NA	1 [Reference]	NA	1 [Reference]	NA
51-70	1.12 (1.03-1.23)^b^	.008	1.20 (1.05-1.36)^b^	.006	1.15 (1.01-1.30)^b^	.04	1.10 (1.01-1.20)^b^	.02	1.08 (0.93-1.26)	.32
71-90	1.20 (1.10-1.31)^b^	<.001	1.27 (1.12-1.44)^b^	<.001	1.21 (1.06-1.37)^b^	.003	1.13 (1.03-1.23)^b^	.007	1.10 (0.95-1.28)	.21
91-100	1.32 (1.18-1.46)^b^	<.001	1.47 (1.26-1.71)^b^	<.001	1.56 (1.34-1.81)^b^	<.001	1.32 (1.18-1.46)^b^	<.001	1.28 (1.06-1.55)^b^	.009
Day										
0-50	1 [Reference]	NA	1 [Reference]	NA	1 [Reference]	NA	1 [Reference]	NA	1 [Reference]	NA-
51-70	0.99 (0.91-1.09)	.88	0.93 (0.81-1.06)	.25	0.92 (0.81-1.04)	.18	1.00 (0.92-1.10)	.96	0.89 (0.76-1.04)	.15
71-90	0.95 (0.86-1.05)	.32	0.89 (0.77-1.03)	.11	0.75 (0.65-0.86)^b^	<.001	0.93 (0.84-1.02)	.14	0.92 (0.78-1.09)	.34
91-100	0.87 (0.77-0.98)^b^	.03	0.91 (0.77-1.09)	.33	0.72 (0.60-0.86)^b^	<.001	0.93 (0.82-1.05)	.23	0.73 (0.58-0.93)^b^	.009
Model 2										
Night										
0-50	1 [Reference]	NA	1 [Reference]	NA	1 [Reference]	NA	1 [Reference]	NA	1 [Reference]	NA
51-70	1.12 (1.03-1.22)^b^	.01	1.21 (1.07-1.38)^b^	.003	1.15 (1.02-1.31)^b^	.03	1.11 (1.01-1.21)^b^	.02	1.09 (0.94-1.28)	.26
71-90	1.19 (1.09-1.30)^b^	<.001	1.27 (1.12-1.45)^b^	<.001	1.20 (1.06-1.37)^b^	.004	1.12 (1.03-1.23)^b^	.009	1.11 (0.95-1.30)	.17
91-100	1.29 (1.16-1.43)^b^	<.001	1.45 (1.24-1.69)^b^	<.001	1.53 (1.31-1.77)^b^	<.001	1.31 (1.18-1.46)^b^	<.001	1.30 (1.08-1.57)^b^	.007
Day										
0-50	1 [Reference]	NA	1 [Reference]	NA	1 [Reference]	NA	1 [Reference]	NA	1 [Reference]	NA
51-70	1.00 (0.91-1.09)	.99	0.93 (0.81-1.06)	.28	0.94 (0.83-1.07)	.36	1.01 (0.93-1.11)	.78	0.88 (0.75-1.03)	.12
71-90	0.97 (0.88-1.07)	.51	0.90 (0.78-1.04)	.15	0.78 (0.67-0.90)^b^	<.001	0.94 (0.85-1.04)	.23	0.94 (0.79-1.11)	.47
91-100	0.89 (0.78-1.01)	.08	0.94 (0.78-1.12)	.48	0.77 (0.64-0.92)^b^	.005	0.94 (0.83-1.07)	.33	0.75 (0.59-0.94)^b^	.01
Model 3										
Night										
0-50	1 [Reference]	NA	1 [Reference]	NA	1 [Reference]	NA	1 [Reference]	NA	1 [Reference]	NA
51-70	1.11 (1.01-1.21)^b^	.03	1.20 (1.06-1.37)^b^	.005	1.15 (1.01-1.31)^b^	.03	1.09 (0.99-1.19)	.07	1.09 (0.93-1.28)	.27
71-90	1.18 (1.08-1.29)^b^	<.001	1.27 (1.11-1.44)^b^	<.001	1.19 (1.05-1.36)^b^	.008	1.10 (1.00-1.20)^b^	.04	1.08 (0.93-1.27)	.31
91-100	1.23 (1.10-1.38)^b^	<.001	1.42 (1.21-1.66)^b^	<.001	1.45 (1.24-1.69)^b^	<.001	1.28 (1.15-1.43)^b^	<.001	1.28 (1.06-1.55)^b^	.01
Day										
0-50	1 [Reference]	NA	1 [Reference]	NA	1 [Reference]	NA	1 [Reference]	NA	1 [Reference]	NA
51-70	1.04 (0.95-1.14)	.44	0.98 (0.85-1.12)	.74	1.00 (0.87-1.14)	.97	1.05 (0.96-1.15)	.33	0.91 (0.77-1.07)	.26
71-90	1.05 (0.95-1.16)	.34	0.97 (0.83-1.12)	.66	0.88 (0.76-1.02)	.10	0.99 (0.90-1.10)	.86	0.99 (0.83-1.18)	.92
91-100	1.02 (0.90-1.17)	.72	1.06 (0.88-1.28)	.55	0.93 (0.77-1.13)	.48	1.02 (0.90-1.16)	.75	0.82 (0.65-1.05)	.11

Abbreviations: HR, hazard ratio; NA, not applicable.

^a^ HRs (95% CIs) adjusted for age, sex, ethnicity, and photoperiod (model 1); additionally adjusted for education, employment, income, and deprivation (model 2); and further adjusted for physical activity, smoking status, alcohol consumption, diet, and urbanicity (model 3). Case numbers by light exposure percentile groups for each model are reported in eTable 5 in Supplement 1.

^b^ *P* < .05.

**Table 3.** Cardiovascular Risks Associated With SD Increases in Night Light Exposure, Including Interactions With Age, Sex, and Polygenic Cardiovascular Disease Risk^a^

Exposure	Coronary artery disease, HR (95% CI)	*P* value	Myocardial infarction, HR (95% CI)	*P* value	Heart failure, HR (95% CI)	*P* value	Atrial fibrillation, HR (95% CI)	*P* value	Stroke, HR (95% CI)	*P* value
Model 3										
Night light	1.05 (1.03-1.08)^b^	<.001	1.07 (1.04-1.11)^b^	<.001	1.08 (1.05-1.11)^b^	<.001	1.05 (1.03-1.08)^b^	<.001	1.05 (1.01-1.09)^b^	.03
Male sex	2.33 (2.17-2.50)^b^	<.001	2.57 (2.31-2.86)^b^	<.001	1.86 (1.68-2.07)^b^	<.001	1.84 (1.72-1.98)^b^	<.001	1.46 (1.29-1.65)^b^	<.001
Age	1.66 (1.58-1.74)^b^	<.001	1.63 (1.52-1.75)^b^	<.001	2.26 (2.09-2.45)^b^	<.001	2.18 (2.07-2.30)^b^	<.001	1.98 (1.81-2.17)^b^	<.001
Model 3 + (night light × sex) + (night light × age)										
Night light	1.10 (1.05-1.16)^b^	<.001	1.14 (1.05-1.23)^b^	.001	1.23 (1.13-1.35)^b^	<.001	1.14 (1.07-1.21)^b^	<.001	1.05 (0.95-1.17)	.34
Male sex	2.54 (2.29-2.82)^b^	<.001	2.77 (2.36-3.24)^b^	<.001	2.16 (1.86-2.52)^b^	<.001	1.94 (1.76-2.15)^b^	<.001	1.49 (1.25-1.77)^b^	<.001
Age	1.68 (1.58-1.80)^b^	<.001	1.68 (1.53-1.85)^b^	<.001	2.44 (2.19-2.73)^b^	<.001	2.30 (2.14-2.47)^b^	<.001	1.98 (1.75-2.24)^b^	<.001
Night light × male sex	0.95 (0.91-0.99)^b^	.02	0.96 (0.90-1.02)	.21	0.92 (0.86-0.98)^b^	.006	0.97 (0.93-1.01)	.14	0.99 (0.91-1.07)	.74
Night light × age	0.99 (0.97-1.02)	.48	0.98 (0.95-1.02)	.34	0.96 (0.92-1.00)^b^	.04	0.97 (0.94-1.00)^b^	.02	1.00 (0.95-1.05)	.98
Model 3 + polygenic risk										
Night light	1.05 (1.03-1.08)^b^	<.001	1.07 (1.04-1.11)^b^	<.001	1.08 (1.05-1.12)^b^	<.001	1.05 (1.03-1.08)^b^	<.001	1.05 (1.01-1.10)^b^	.01
Polygenic risk	1.14 (1.11-1.18)^b^	<.001	1.14 (1.08-1.20)^b^	<.001	1.20 (1.14-1.26)^b^	<.001	1.16 (1.12-1.20)^b^	<.001	1.13 (1.06-1.20)^b^	<.001
Model 3 + (polygenic risk × night light)										
Night light	1.05 (1.01-1.08)^b^	.006	1.04 (0.99-1.09)	.17	1.06 (1.01-1.12)^b^	.01	1.04 (1.00-1.07)^b^	.05	1.01 (0.95-1.07)	.75
Polygenic risk	1.14 (1.08-1.20)^b^	<.001	1.08 (1.00-1.16)	.06	1.17 (1.09-1.26)^b^	<.001	1.13 (1.08-1.19)^b^	<.001	1.06 (0.97-1.16)	.19
Night light × polygenic risk	1.00 (0.98-1.03)	.80	1.03 (1.00-1.07)	.05	1.01 (0.98-1.05)	.42	1.02 (0.99-1.04)	.19	1.04 (1.00-1.08)	.06

Abbreviation: HR, hazard ratio.

^a^ HRs (95% CIs) are adjusted for model 3 covariates (age, sex, ethnicity, photoperiod, education, employment, income, deprivation, physical activity, smoking status, alcohol consumption, diet, and urbanicity). HRs for age and polygenic risk are per SD, and HRs for night light are per 1-unit increase in log-transformed light exposure.

^b^ *P* < .05.

Brighter night light was associated with higher cardiovascular disease risks following separate adjustments of model 3 for preexisting diabetes, hypertension, high body mass index, high cholesterol ratio, chronotype, sleep efficiency, short or long sleep, and exclusion of shift workers (eTables 6-10 in Supplement 1). Associations of night light with cardiovascular risks were attenuated but remained statistically significant for all outcomes except stroke, which was attenuated below statistical significance after adjustment for short sleep and high cholesterol ratio. Associations of night light with cardiovascular risks did not differ by chronotype (eTable 11 in Supplement 1). Models 1 to 3 were also tested after (1) excluding participants with any of the 5 cardiovascular outcomes before light tracking, rather than applying separate exclusions for each cardiovascular outcome, and (2) advancing the observation period commencement to the date of baseline assessment (March 2006 to October 2010), rather than the date of light tracking. Associations of brighter nights with higher cardiovascular risks were robust to both analyses (eTables 12-13 in Supplement 1).

Brighter day light exposure was associated with lower risks of coronary artery disease, heart failure, and stroke in models 1 and 2. Those with the brightest days (91st-100th percentiles) had lower risk of coronary artery disease (model 1: HR, 0.87; 95% CI, 0.77-0.98), heart failure (model 1: HR, 0.72; 95% CI, 0.60-0.86; model 2: 0.77; 95% CI, 0.64-0.92), and stroke (model 1: HR, 0.73; 95% CI, 0.58-0.93; model 2: HR, 0.75; 95% CI, 0.59-0.94) compared with those with darker days (0-50th percentiles). Point estimates for brighter day light and risk of myocardial infarction and atrial fibrillation were not statistically significant. Brighter day light exposure was not associated with risk of any cardiovascular disease in model 3, which included additional adjustment for lifestyle factors. However, after excluding physical activity from model 3, brighter day light was associated with heart failure and stroke (eTables 8 and eTable 10 in Supplement 1).

### Night Light Exposure and Cardiovascular Risk, According to Age, Sex, and Genetic Susceptibility

The magnitude of associations of night light exposure with heart failure, coronary artery disease, and atrial fibrillation risk varied according to participant age and/or sex (Table 3). Brighter night light had a larger-magnitude association with adjusted risk for heart failure in females (*P* for interaction = .006) and in younger individuals (*P *for interaction = .04). Brighter night light diminished the protective association of being female with risk of heart failure, such that females exposed to bright night light had similar heart failure risks to males exposed to bright night light (eFigure 1 in Supplement 1). For risk of coronary artery disease, the magnitude of the association of brighter night light with higher adjusted risk was larger in females than males (*P *for interaction = .02) (eFigure 2 in Supplement 1). For risk of atrial fibrillation, the association of brighter night light with higher adjusted risk was greater in younger individuals (*P *for interaction = .02) (eFigure 3 in Supplement 1). The magnitude of the associations of brighter night light with myocardial infarction and stroke risk did not vary with age or sex.

Associations of night light with higher cardiovascular risk were robust to including polygenic risk for cardiovascular disease alongside model 3 covariates. No significant interactions of night light exposure with polygenic risk were observed for any cardiovascular outcome (Table 3).

## Discussion

In this cohort study, across approximately 13 million hours of personal light exposure data, and approximately 700 000 person-years of follow-up, individuals exposed to higher levels of night light had higher risks for incident coronary artery disease, myocardial infarction, heart failure, atrial fibrillation, and stroke. These associations of night light with cardiovascular disease risk were robust to adjustment for cardiovascular risk factors including physical activity, diet, sleep, and genetic susceptibility. These findings support night light exposure as an important risk factor for adverse cardiovascular health.

The observed associations of brighter night light with higher cardiovascular disease risks are consistent with previous studies of outdoor night light. We observed a 23% to 32% higher risk of coronary artery disease, and a 45% to 56% higher risk of myocardial infarction for people with the brightest nights (91st to 100th percentiles), compared with those with the darkest nights (0 to 50th percentiles). A previous cohort study that used satellite data to define light exposure found that people with the brightest outdoor nights (top 20%) had a 7% to 23% greater risk of coronary heart disease, compared with those with the darkest outdoor nights (lowest 20%).^24^ We also observed a 28% to 30% higher risk of stroke, whereas a previous satellite data study found a 26% to 43% higher risk of stroke for people with the brightest outdoor nights (top 25%), compared with those with darker outdoor nights (lowest 25%).^25^ Furthermore, we observed a 45% to 56% higher risk of heart failure, and a 28% to 32% higher risk of atrial fibrillation, for people with the brightest nights. To our knowledge, no previous large-scale studies have assessed whether light exposure is associated with heart failure or atrial fibrillation. Our findings are consistent with higher prevalence of cardiovascular risk factors in people with brighter nights, observed in smaller cohorts with objective light data.^26,27,28^ Finally, our findings are consistent with higher cardiovascular risks observed in rotating shift workers,^16,17,18,19,20^ a population that experiences frequent exposure to bright light during the biological night.

The observed higher risks of cardiovascular diseases in people with brighter nights may be explained by the disruptive effect of night light on circadian rhythms,^21,22,23^ which can lead to dysregulation of various cardiovascular and metabolic mechanisms. First, circadian disruption is strongly implicated in impaired glucose tolerance^12,47^ and type 2 diabetes,^33,48,49^ which are significant risk factors for endothelial dysfunction and atherosclerosis. Second, circadian disruption may promote hypercoagulability,^9^ increasing risks of thromboembolic events and subsequent ischemia, particularly in people with atherosclerosis or atrial fibrillation. Third, circadian disruption can cause higher average 24-hour blood pressure,^11,50,51^ potentially increasing risks for vascular endothelial damage and myocardial hypertrophy. Finally, central circadian disruption may increase the risk of cardiac arrhythmia, due to conflicting inputs to the sinoatrial and atrioventricular nodes from the central circadian clock and cardiomyocyte clocks.^8^ Together, these mechanisms may explain the observed higher risks of cardiovascular diseases with brighter night light exposure.

The associations of night light with heart failure, coronary artery disease, and atrial fibrillation risks differed according to age and/or sex. We observed larger-magnitude associations of night light with risks of heart failure and coronary artery disease in women. These findings are consistent with previous research showing that exposure to shift work, which causes circadian disruption, predicts higher risk of heart failure in women compared with men.^18^ Greater sensitivity of the circadian system to bright light has also been observed in women, compared with men.^52^ We observed larger-magnitude associations of night light exposure with heart failure and atrial fibrillation risks for younger individuals. This finding may be attributable to attenuated circadian light sensitivity in older individuals.^53^

The dose-response associations of brighter nights with higher risk of cardiovascular diseases were robust after accounting for genetic susceptibility for these diseases. This finding is important due to potential confounding by gene-environment correlation.^54,55^ For example, greater genetic susceptibility for cardiovascular diseases could influence both night light exposure behavior and risk for developing a cardiovascular disease. The association of night light exposure with cardiovascular outcomes was independent of polygenic risk for cardiovascular diseases, indicating that gene-environment correlation was an unlikely factor underlying the observed associations.

Exposure to night light is a plausible proxy for sleep duration, which has been associated with risk of cardiovascular diseases.^56,57^ However, observed associations of brighter nights with higher risks of coronary artery disease, myocardial infarction, heart failure, and atrial fibrillation were independent of short and long sleep and sleep efficiency. The associations of the brightest night light with cardiovascular risks were attenuated but remained statistically significant following adjustment for short sleep duration, indicating that short sleep explained some, but not all, of the observed associations. These findings are consistent with experiments demonstrating sleep-independent effects of light on circadian regulation of factors known to influence cardiovascular health, such as the secretion of glucagon-like peptide-1.^47^

### Strengths and Limitations

This, to our knowledge, is the largest known study of prospective associations of personal light exposure with cardiovascular disease risk. Results were derived from approximately 13 million hours of personal light exposure data from wrist-worn sensors, coupled with health records collected across a subsequent 9.5-year period, in approximately 89 000 individuals. Detailed sociodemographic and lifestyle information, objective sleep and physical activity data, and genetic susceptibility data were available.

However, this study has several limitations. First, whether these findings generalize is not yet clear. The UK Biobank cohort is predominantly White (97%), and overrepresents individuals with higher education levels, higher income, women (57%), and healthier individuals.^58^ Second, longer-term within-individual light tracking would improve our estimation of cardiovascular-light associations, above the single week of light tracking used here. However, light exposures displayed within-individual consistency in a subsample of UK Biobank participants with repeated 1-week measures.^35^ Third, information about light exposure sources was not available, meaning we could not adjust for behavioral correlates of night light exposure (eg, light from stimulating digital content). Fourth, some covariates included in analyses may be on causal pathways between night light exposure and cardiovascular risks (eg, physical activity). Fifth, some covariates were collected prior to light tracking and may be subject to change over time. Sixth, these findings were observational and did not capture the causal relationship of night light with cardiovascular disease risk. Long-term circadian-informed lighting interventions for reducing cardiovascular disease risk are needed.

## Conclusions

Cardiovascular diseases are the leading cause of global morbidity and mortality.^59^ Current preventive recommendations include maintaining a healthy diet, attaining adequate physical activity, and avoiding alcohol and tobacco.^60^ To our knowledge, this is the first study of personal light exposure patterns and incident cardiovascular diseases, indicating night light as an important new risk factor. Our findings demonstrate that, additional to current recommendations, avoiding night light is a promising target for preventing cardiovascular diseases.

## Notes

Supplement 1. eTable 1. UK Biobank protocol documentation eMethods. eTable 2. Covariates included in statistical analyses eTable 3. Participant characteristics for the total analysis sample, and for sub-groups without each cardiovascular outcome prior to light tracking eTable 4. Cases of cardiovascular diseases by light exposure percentile groups, by subsets included in Models 1-3 eTable 5. Participant characteristics by light exposure percentiles, by day and night eTable 6. Relationships of day and night light with coronary artery disease, adjusted for pre-existing cardiometabolic health, sleep, and excluding shift workers eTable 7. Relationships of day and night light with myocardial infarction, adjusted for pre-existing cardiometabolic health, sleep, and excluding shift workers eTable 8. Relationships of day and night light with heart failure, adjusted for pre-existing cardiometabolic health, sleep, and excluding shift workers eTable 9. Relationships of day and night light with atrial fibrillation, adjusted for pre-existing cardiometabolic health, sleep, and excluding shift workers eTable 10. Relationships of day and night light with stroke, adjusted for pre-existing cardiometabolic health, sleep, and excluding shift workers eTable 11. Interaction of chronotype and night light exposure for cardiovascular risks eTable 12. Risk of cardiovascular outcomes across Models 1-3 after excluding participants with any cardiovascular disease prior to light tracking eTable 13. Risk of cardiovascular outcomes across Models 1-3 from UK Biobank enrolment (commencing March 2006) to study administrative endpoint (November 2022) eFigure 1. Relationship of night light exposure with risk of heart failure, according to participant age and sex. eFigure 2. Relationship of night light exposure with risk of coronary artery disease, according to participant age and sex. eFigure 3. Relationship of night light exposure with risk of atrial fibrillation, according to participant age and sex. eReferences.

Supplement 2. Data Sharing Statement
