# Supplementary material for: Light Exposure at Night and Cardiovascular Disease Incidence
Source: JAMA Netw Open. 2025 Oct 23;8(10):e2539031. doi: 10.1001/jamanetworkopen.2025.39031 (PMC12550636; doi:10.1001/jamanetworkopen.2025.39031)
Supplement: Supplement 1. — eTable 1. UK Biobank protocol documentation eMethods. eTable 2. Covariates included in statistical analyses eTable 3. Participant characteristics for the total analysis sample, and for sub-groups without each cardiovascular outcome prior to light tracking eTable 4. Cases of cardiovascular diseases by light exposure percentile groups, by subsets included in Models 1-3 eTable 5. Participant characteristics by light exposure percentiles, by day and night eTable 6. Relationships of day and night light with coronary artery disease, adjusted for pre-existing cardiometabolic health, sleep, and excluding shift workers eTable 7. Relationships of day and night light with myocardial infarction, adjusted for pre-existing cardiometabolic health, sleep, and excluding shift workers eTable 8. Relationships of day and night light with heart failure, adjusted for pre-existing cardiometabolic health, sleep, and excluding shift workers eTable 9. Relationships of day and night light with atrial fibrillation, adjusted for pre-existing cardiometabolic health, sleep, and excluding shift workers eTable 10. Relationships of day and night light with stroke, adjusted for pre-existing cardiometabolic health, sleep, and excluding shift workers eTable 11. Interaction of chronotype and night light exposure for cardiovascular risks eTable 12. Risk of cardiovascular outcomes across Models 1-3 after excluding participants with any cardiovascular disease prior to light tracking eTable 13. Risk of cardiovascular outcomes across Models 1-3 from UK Biobank enrolment (commencing March 2006) to study administrative endpoint (November 2022) eFigure 1. Relationship of night light exposure with risk of heart failure, according to participant age and sex. eFigure 2. Relationship of night light exposure with risk of coronary artery disease, according to participant age and sex. eFigure 3. Relationship of night light exposure with risk of atrial fibrillation, according to participant age and sex. eReferences. [file jamanetwopen-e2539031-s001.pdf]

Supplemental Online Content

Windred DP, Burns AC, Rutter MK, et al. Light exposure at night and cardiovascular disease incidence. *JAMA Netw. Open*. 2025;8(10):e2539031. doi:10.1001/jamanetworkopen.2025.39031

eTable 1. UK Biobank protocol documentation

eMethods.

eTable 2. Covariates included in statistical analyses

eTable 3. Participant characteristics for the total analysis sample, and for sub-groups without each cardiovascular outcome prior to light tracking

eTable 4. Cases of cardiovascular diseases by light exposure percentile groups, by subsets included in Models 1-3

eTable 5. Participant characteristics by light exposure percentiles, by day and night

eTable 6. Relationships of day and night light with coronary artery disease, adjusted for pre-existing cardiometabolic health, sleep, and excluding shift workers

eTable 7. Relationships of day and night light with myocardial infarction, adjusted for pre-existing cardiometabolic health, sleep, and excluding shift workers

eTable 8. Relationships of day and night light with heart failure, adjusted for pre-existing cardiometabolic health, sleep, and excluding shift workers

eTable 9. Relationships of day and night light with atrial fibrillation, adjusted for pre-existing cardiometabolic health, sleep, and excluding shift workers

eTable 10. Relationships of day and night light with stroke, adjusted for pre-existing cardiometabolic health, sleep, and excluding shift workers

eTable 11. Interaction of chronotype and night light exposure for cardiovascular risks

eTable 12. Risk of cardiovascular outcomes across Models 1-3 after excluding participants with any cardiovascular disease prior to light tracking

eTable 13. Risk of cardiovascular outcomes across Models 1-3 from UK Biobank enrolment (commencing March 2006) to study administrative endpoint (November 2022)

eFigure 1. Relationship of night light exposure with risk of heart failure, according to participant age and sex. This figure captures the marginal effects of light exposure on heart failure risk at specified ages, for males and females, and is derived from Table 3: Model 3 + Night light*Sex + Night light*Age.

eFigure 2. Relationship of night light exposure with risk of coronary artery disease, according to participant age and sex. This figure captures the marginal effects of light exposure on coronary artery disease risk at specified ages, for males and females, and is derived from Table 3: Model 3 + Night light*Sex + Night light*Age

eFigure 3. Relationship of night light exposure with risk of atrial fibrillation, according to participant age and sex. This figure captures the marginal effects of light exposure on atrial fibrillation risk at specified ages, for males and females, and is derived from Table 3: Model 3 + Night light*Sex + Night light*Age

eReferences.

This supplemental material has been provided by the authors to give readers additional information about their work.

© 2025 Windred DP et al. *JAMA Network Open*.

eTable 1. UK Biobank protocol documentation

Documentation	UK Biobank Website Link
Invite to participate	https://biobank.ctsu.ox.ac.uk/crystal/refer.cgi?id=100253
Participant instructions (Axivity AX3 device)	https://biobank.ndph.ox.ac.uk/showcase/refer.cgi?id=141141
Collection and processing (Axivity AX3 device)	https://biobank.ndph.ox.ac.uk/showcase/refer.cgi?id=131600
‘First occurrence’ cardiovascular outcomes	https://biobank.ndph.ox.ac.uk/showcase/refer.cgi?id=593
‘Algorithmically defined’ cardiovascular outcomes	https://biobank.ndph.ox.ac.uk/showcase/refer.cgi?id=460
Death register	https://biobank.ctsu.ox.ac.uk/crystal/refer.cgi?id=115559
Ethics	https://www.ukbiobank.ac.uk/learn-more-about-uk-biobank/about-us/ethics
Reception and consent (assessment centre visit)	https://biobank.ndph.ox.ac.uk/showcase/refer.cgi?id=100230<br>https://biobank.ndph.ox.ac.uk/showcase/ukb/docs/Reception.pdf
Physical measurements (assessment centre visit)	https://biobank.ndph.ox.ac.uk/showcase/refer.cgi?id=100225<br>https://biobank.ndph.ox.ac.uk/showcase/refer.cgi?id=5636<br>https://biobank.ndph.ox.ac.uk/showcase/refer.cgi?id=1227

© 2025 Windred DP et al. *JAMA Network Open*.

eMethods.

Cardiovascular outcomes

Coronary artery disease was defined according to first occurrence outcomes and operations. First occurrences were defined according to ICD-10 codes for acute myocardial infarction (I21), subsequent myocardial infarction (I22), complications following myocardial infarction (I23), acute ischaemic heart disease (I24), and chronic ischaemic heart disease (I25). Operations were defined according to OPCS4 codes K40-46, K49, K50, and K75, or self-reported coronary angioplasty, coronary artery bypass, or triple heart bypass. Atrial fibrillation was defined according to first occurrence of ICD-10 code I48, or according to OPCS codes K62.1, K62.2, or K62.3.

Day and night light exposure

Factor analysis was applied to extract time windows where light exposure patterns exhibited clustered variance, according to the methodology reported in our previous work.[1] In short, 48 half-hour light exposure bins, representing all clock times (e.g., 00:30 to 01:00), were used to extract night and day light factors. Varimax rotation with factor loading ≥0.5 was applied, and cumulative proportion of variance explained was 0.56. Factors were internally consistent (Cronbach's α=.98 and α=.93, respectively), and weakly positively correlated (rs=0.10, p<.0001).

Night light exposure (00:30-06:00) was split into four percentile groups, with category boundaries as follows: <1.2 lx (0-50%); ≥1.2 & <6.2 lx (50-70%); ≥6.2 & < 48.3 lx (70-90%); and ≥48.3 lx (90-100%). Similarly, day light exposure (07:30-20:30) was split into four percentile groups, with category boundaries as follows: <991 lx (0-50%); ≥991 & <1750 lx (50-70%); ≥1750 & <3140 lx (70-90%); and ≥3140 lx (90-100%).

Light sensor reliability

We have previously tested a sample of Axivity AX3 devices under reference lighting conditions, allowing for approximating illuminance (lux) from device data.[1] Here, we additionally report device reliability from this testing data. We calculated intra-class correlation coefficients (ICCs) to determine absolute agreement between devices within various reference illuminance ranges. ICC values were calculated using two-way random effects models (*irr* package, R 4.5.0.). ICC values close to one indicated excellent consistency between devices, as follows: ICC [95%CI] 0-25 lux = 0.92 [0.83-0.97]; ICC [95%CI] 0-50 lux = 0.93 [0.86-0.97]; ICC [95%CI] 0-100 lux = 0.94 [0.87-0.97]; ICC [95%CI] 0-3000 lux = 0.95 [0.92-0.97].

Polygenic risk scores

Polygenic risk scores (PRS) for CAD[2], myocardial infarction[2], heart failure[3], atrial fibrillation[2], and stroke[4] was constructed using PRS-continuous shrinkage (PRS-CS).[5] PRS–CS uses a Bayesian framework and a continuous shrinkage prior on SNP effect sizes while modelling linkage disequilibrium to improve polygenic prediction. These PRS were then scored in UK Biobank participants using PLINK 2[6] as the weighted sum of the effect alleles, using the following formula:

$$S_i = \sum_{j=1}^{M} \hat{\beta}_j g_{ij}$$

where S_i is the polygenic score for individual *i*, $\hat{\beta}_j$ is the weighted additive effect of the effect allele at SNP *j*, and g_{ij} is the genotype for individual *i* at SNP *j*.

Genetic ancestry definition and principal components of ancestry

European ancestry classification was completed using the Human Genome Diversity Project-1000 Genomes (HGDP-1KG) harmonized reference dataset.[7] The HGDP-1KG is a high quality dataset of 4,094 whole genomes from labelled diverse continental populations. Principal components analysis (PCA) was performed on unrelated (KING kinship coefficient < 0·125) individuals after pruning variants (500kb window, r2 = 0·01) to extract the top 10 PCs of ancestry.[8-10] We then projected individuals from the UK Biobank onto the HGDP-1KG PC space and trained a random forest classifier given continental ancestry labels from the HGDP-1KG cohort to assign ancestry to UK Biobank individuals based on their top 10 PC scores. The minimum random forest probability for assignment to

© 2025 Windred DP et al. *JAMA Network Open*.

a particular ancestry group was 0·5 and we completed 20 iterations of this model. An individual was assigned to the European ancestry group and included in genetic cox models if 20/20 iterations assigned them to the European ancestry, otherwise individuals were excluded as non-European or admixed. Finally, PCA was completed within European UKB individuals to extract the top 5 PCs of ancestry for inclusion as population stratification covariates in genetic cox models.

Model implementation

Cox proportional hazards models were executed in R (version 4.4.1.), using the ‘survival’ package. The following is an example of model syntax:

coxph(Surv(years, myocardial_infarction_diagnosis) ~ light_night + light_day + age + sex + ethnicity, data=data)

Contrasts were set using the default ‘contr.treatment’ setting, for all categorical variables. The assumption of proportional hazards was assessed using the ‘cox.zph’ function.

Models assessing interactions of night light with age, sex, and genetic susceptibility as predictors of cardiovascular risks were implemented. In these models, night light was treated as a continuous variable, transformed according to the following equation:

transformed_night_light = log(night_light + 1)

Figures for each interaction model represented marginal effects and were generated using the ‘sjPlot’ package (‘plot_model’ function, type = ‘pred’).

© 2025 Windred DP et al. *JAMA Network Open*.

eTable 2. Covariates included in statistical analyses

Covariate	UKB ID(s)	Description	Model variable
Age	21003	Obtained from NHS Primary Care Trust registries and confirmed with participants at assessment centre visit.	Continuous
Sex	31	Obtained from NHS Primary Care Trust registries and confirmed with participants at assessment centre visit.	Categorical: male, female
Ethnicity*	21000	Self-reported ethnic group, categories defined by the UK Biobank team: 'Asian' (Indian, Pakistani, Bangladeshi, or Other); 'Black' (Caribbean, African, or Other); 'Chinese'; 'Mixed' (White and Black Caribbean, White and Black African, White and Asian, or Other); 'White' (British, Irish, or Other); 'Other ethnic group'; do not know; and prefer not to say	Categorical: white, other
Education	6138	University, A Levels, O Levels, CSE, NVQ/HND/HNC, other, none, prefer not to say	Categorical: university (referent), other non-university (selection of any education category except university), none
Employment status	6142	Paid employment, unemployed, retired, home/family caretaker, unable to work, volunteer, student, other	Categorical: paid employment, other categories
Income	738	Yearly household income: <£18,000, £18,000-£29,900, £30,000-£51,900, £52,000-£100,000, >£100,000, do not know, prefer not to say	Categorical: <£18k, £18k-£29.9k, £30k-£51.9k, £52k-£100k, >£100k (referent), and unknown
Deprivation	189	Average home ownership, car ownership, household overcrowding, and employment rate of a participant's local area. Derived using national census data at time of recruitment.	Continuous, included as recorded
Physical activity	90012	Accelerometer device average acceleration across one week of data collection	Continuous, included as recorded
Smoking status	20116	Smoking status: never, previous, current, prefer not to say	Categorical: current, previous, never (referent)
Alcohol consumption	1558	Alcohol intake frequency: daily, 3-4 times per week, 1-2 times per week, 1-3 times per month, special occasions only, never, prefer not to say	Continuous, days per week consuming alcohol: daily = 7, '3-4 times per week' = 3.5, 1-2 times per week = 1.5, 1-3 times per month = 2*12/365.25*7, special occasions only = 1*12/365.25*7, never = 0
Healthy diet	1289, 1299, 1309, 1319, 1329, 1339, 1349, 1359, 1369, 1379, 1389, 1408, 1418, 1428, 1438, 1448, 1458, 1468, 2654, 3680, 6144	Typical dietary intake. 10 nutritional intake criteria for cardiometabolic health were included, as reported previously[11]	Categorical: healthy, unhealthy. Classified as healthy if ≥5 out of 10 dietary criteria were met.

© 2025 Windred DP et al. *JAMA Network Open*.

Urbanicity	20118	Population density of participants' local area, attained from the UK Office for National Statistics.	Categorical: urban (population ≥ 10,000), rural (population < 10,000)
Shift work	826, 3426, 6142	Participants' work involves shift work or night shift work: never, sometimes, usually, always, do not know, prefer not to say	Categorical: shift-worker ('sometimes', 'usually' or 'always' for either 'shift work' or 'night shift work'), non-shift worker ('never/rarely', or 'unemployed').
BMI	21001	Weight (kg) / height $(m)^2$	Categorial: high (BMI > 30), low (BMI ≤ 30)
Cholesterol ratio	30760, 30780, 30870	Blood biochemistry assays for high- and low-density lipoprotein, and triglycerides	Categorical: high (cholesterol ratio >3.75 for males or >3.00 for females), low (cholesterol ratio ≤3.75 for males or ≤3.00 for females). Calculated as cholesterol ratio = (HDL + LDL + 0.2*triglycerides)/HDL
Hypertension	4080, 4079, 131286, 131288, 131290, 131292, 131294	Physical measurement at assessment centre visit, two systolic and diastolic readings, averaged, or first occurrence of hypertension (ICD-10 codes I10-13, I15).	Categorical: high (systolic > 140, diastolic > 90, or diagnosed hypertension prior to light tracking), low (systolic ≤ 140, diastolic ≤ 90, or no diagnosed hypertension prior to light tracking)
Diabetes	2443, 130706, 130708	Diabetes diagnosed by a doctor: yes, no, do not know, prefer not to say, Diabetes diagnosed according to ICD-10 codes E10 or E11.	Categorical: diabetes, no diabetes
Photoperiod	N/A	Calculated from date of light tracking and coordinates of 53.4808° N, 2.2426° W (Manchester), using 'getSunlightTimes()' in the 'suncalc' package in R.	Continuous
Chronotype	1180	Self-reported as 'definitely a morning person', 'more a morning than evening person', 'more an evening than morning person', or 'definitely an evening person'	Categorical: morning (referent; definitely morning and mostly morning), evening (mostly evening, definitely evening)

*Ethnicity was included in analyses due to known differences in adverse cardiovascular outcomes between people who identify as 'White' ethnicity and other ethnic groups[12]

© 2025 Windred DP et al. *JAMA Network Open*.

eTable 3. Participant characteristics for the total analysis sample, and for sub-groups without each cardiovascular outcome prior to light tracking

	No exclusion	Coronary artery disease	Myocardial infarction	Heart failure	Atrial fibrillation	Stroke
Age						
M±SD	62.4±7.8	62.1±7.8	62.3±7.8	62.4±7.8	62.2±7.8	62.3±7.8
Range	43.5 to 79.0	43.5 to 79.0	43.5 to 79.0	43.5 to 79.0	43.5 to 79.0	43.5 to 79.0
Sex (% male, N)	43.1 (38321)	41.6 (35205)	42.3 (36748)	42.9 (37836)	42.4 (36558)	42.9 (37661)
Ethnicity (% white, N)	97.0 (85923)	97.0 (81838)	97.0 (83978)	97.0 (85275)	96.9 (83332)	97.0 (84779)
Employment status (% employed, N)	62.1 (54843)	63.1 (53102)	62.6 (53994)	62.3 (54573)	62.7 (53715)	62.4 (54374)
Income						
% <£18k, N	13.0 (11502)	12.6 (10615)	12.8 (11057)	13.0 (11350)	12.9 (11059)	12.9 (11240)
% £18-29.9k, N	21.7 (19203)	21.5 (18058)	21.6 (18647)	21.7 (19009)	21.6 (18506)	21.7 (18899)
% £30-51.9k, N	26.0 (22912)	26.1 (21946)	26.0 (22461)	26.0 (22782)	26.0 (22256)	26.0 (22663)
% £52-100k, N	22.8 (20174)	23.3 (19561)	23.0 (19882)	22.9 (20081)	23.0 (19735)	23.0 (19997)
% >£100k, N	6.7 (5891)	6.8 (5723)	6.7 (5814)	6.7 (5872)	6.7 (5750)	6.7 (5841)
Education						
% other, N	48.3 (42519)	48.2 (40388)	48.3 (41519)	48.3 (42204)	48.4 (41293)	48.3 (41915)
% university/college, N	43.4 (38176)	43.9 (36843)	43.7 (37556)	43.4 (37952)	43.5 (37123)	43.5 (37775)
Townsend Deprivation Index						
M±SD	-1.76±2.80	-1.76±2.80	-1.76±2.80	-1.76±2.80	-1.75±2.80	-1.76±2.79
Range	-6.26 to 10.5	-6.26 to 10.5	-6.26 to 10.5	-6.26 to 10.5	-6.26 to 10.5	-6.26 to 10.5
Smoking						
% previous, N	36.1 (31964)	35.4 (29881)	35.7 (30924)	35.9 (31629)	35.7 (30748)	35.9 (31447)
% current, N	6.8 (6057)	6.8 (5713)	6.8 (5845)	6.8 (6005)	6.9 (5911)	6.8 (5956)
Alcohol (M±SD, days per week)	2.98±2.50	2.97±2.50	2.98±2.50	2.98±2.50	2.97±2.49	2.98±2.50
Urbanicity (% >10,000 population, N)	84.1 (74033)	84.1 (70530)	84.1 (72377)	84.1 (73486)	84.2 (71899)	84.1 (73089)
Physical activity						
M±SD	28.1±8.1	28.3±8.1	28.2±8.1	28.2±8.1	28.2±8.1	28.2±8.1
Range	4.8 to 69.4	4.8 to 69.4	4.8 to 69.4	4.83to 69.4	4.8 to 69.4	4.8 to 69.3
Diet score (% healthy, N)	25.8 (22258)	25.7 (21194)	25.8 (21779)	25.8 (22096)	25.7 (21544)	25.8 (21977)

© 2025 Windred DP et al. *JAMA Network Open*.

eTable 4. Cases of cardiovascular diseases by light exposure percentile groups, by subsets included in Models 1-3

			Coronary artery disease	Myocardial infarction	Heart failure	Atrial fibrillation	Stroke
		Percentile	Cases % (N)	Cases % (N)	Cases % (N)	Cases % (N)	Cases % (N)
Model 1	Night	0-50% (ref.)	4.12 (1738)	1.77 (766)	1.87 (823)	4.15 (1786)	1.34 (584)
		50-70%	4.43 (747)	2.06 (356)	1.97 (347)	4.27 (735)	1.33 (232)
		70-90%	4.73 (799)	2.19 (379)	2.06 (362)	4.37 (751)	1.36 (237)
		90-100%	5.24 (442)	2.53 (219)	2.65 (233)	5.16 (444)	1.60 (140)
	Day	0-50% (ref.)	4.26 (1797)	1.92 (833)	2.02 (886)	4.15 (1786)	1.39 (608)
		50-70%	4.48 (757)	1.92 (333)	2.06 (362)	4.40 (757)	1.30 (228)
		70-90%	4.54 (767)	1.99 (345)	1.85 (326)	4.32 (742)	1.40 (245)
		90-100%	4.80 (405)	2.41 (209)	2.17 (191)	5.01 (431)	1.28 (112)
Model 2	Night	0-50% (ref.)	4.11 (1703)	1.74 (741)	1.84 (796)	4.14 (1750)	1.32 (566)
		50-70%	4.42 (734)	2.06 (351)	1.97 (340)	4.28 (723)	1.32 (227)
		70-90%	4.68 (777)	2.16 (367)	2.03 (351)	4.34 (734)	1.34 (231)
		90-100%	5.15 (427)	2.47 (210)	2.60 (225)	5.12 (433)	1.59 (137)
	Day	0-50% (ref.)	4.24 (1758)	1.91 (812)	1.98 (857)	4.14 (1749)	1.38 (591)
		50-70%	4.45 (738)	1.89 (322)	2.04 (352)	4.40 (743)	1.27 (218)
		70-90%	4.52 (750)	1.96 (333)	1.83 (317)	4.31 (728)	1.41 (242)
		90-100%	4.76 (395)	2.37 (202)	2.15 (186)	4.97 (420)	1.28 (110)
Model 3	Night	0-50% (ref.)	4.11 (1644)	1.73 (710)	1.83 (763)	4.17 (1700)	1.33 (550)
		50-70%	4.40 (705)	2.05 (336)	1.96 (327)	4.24 (692)	1.34 (222)
		70-90%	4.73 (758)	2.15 (354)	2.03 (339)	4.30 (701)	1.34 (222)
		90-100%	5.17 (414)	2.51 (206)	2.61 (218)	5.18 (422)	1.65 (137)
	Day	0-50% (ref.)	4.23 (1692)	1.90 (779)	1.96 (819)	4.12 (1678)	1.39 (575)
		50-70%	4.43 (710)	1.91 (313)	2.03 (339)	4.43 (722)	1.29 (214)
		70-90%	4.57 (731)	1.95 (320)	1.85 (308)	4.34 (707)	1.42 (235)
		90-100%	4.85 (388)	2.36 (194)	2.17 (181)	5.00 (408)	1.29 (107)

© 2025 Windred DP et al. *JAMA Network Open*.

eTable 5. Participant characteristics by light exposure percentiles, by day and night

	Night light exposure percentile				Day light exposure percentile			
	**0-50%**	**50-70%**	**70-90%**	**90-100%**	**0-50%**	**50-70%**	**70-90%**	**90-100%**
BMI (% ≥30, N)	16.9 (7481)	19.8 (3519)	22.2 (3934)	26.0 (2305)	19.7 (8740)	19.9 (3523)	19.2 (3404)	17.7 (1572)
Diabetes (%, N)	3.91 (1739)	4.51 (802)	5.36 (953)	6.03 (536)	4.70 (2089)	4.64 (825)	4.37 (777)	3.81 (339)
Hypertension (%, N)	26.3 (11707)	26.7 (4739)	27.6 (4910)	30.8 (2736)	27.3 (12141)	26.7 (4741)	26.8 (4774)	27.4 (2436)
High cholesterol ratio* (%, N)	65.8 (25125)	66.8 (10233)	66.3 (10127)	68.1 (5253)	66.5 (25415)	66.9 (10193)	66.0 (10125)	65.1 (5005)
Sleep duration (% <6 h, N)	12.1 (5189)	20.6 (3536)	27.2 (4665)	40.0 (3418)	20.1 (8553)	19.4 (3335)	19.2 (3295)	18.9 (1625)
Sleep duration (% <9 h, N)	1.56 (665)	1.82 (312)	0.671 (115)	0.644 (55)	1.48 (631)	1.31 (225)	1.11 (190)	1.17 (101)
Sleep efficiency (range:0-100; M±SD)	89.4±4.90	89.2±5.11	89.0±5.22	88.6±5.83	89.2±5.15	89.2±5.13	89.2±5.07	89.1±5.01
Shift worker (%, N)	6.61 (2918)	8.84 (1560)	9.81 (1730)	10.10 (894)	8.54 (3767)	8.35 (1473)	7.32 (1293)	6.45 (569)
Chronotype								
Morning (%, N)	67.5 (26649)	62.9 (9980)	59.7 (9520)	51.9 (4184)	62.1 (24619)	63.7 (10100)	64.4 (10258)	67.7 (5356)
Evening (%, N)	32.5 (12810)	37.1 (5889)	40.3 (6421)	48.1 (3870)	37.9 (14997)	36.3 (5762)	35.6 (5671)	32.3 (2560)

*High cholesterol ratio was defined as cholesterol ratio >3.75 for males or >3.00 for females

© 2025 Windred DP et al. *JAMA Network Open*.

eTable 6. Relationships of day and night light with coronary artery disease, adjusted for pre-existing cardiometabolic health, sleep, and excluding shift workers

		Percentile	Cases % (N)	HR [95% CI]	p-value
Model 3 + BMI	Night	0-50% (ref.)	4.1 (1639)	-	-
N = 79908		50-70%	4.39 (702)	1.09 [1.00-1.19]	0.05
		70-90%	4.73 (756)	1.15 [1.06-1.26]*	0.001
		90-100%	5.16 (412)	1.19 [1.06-1.33]*	0.002
	Day	0-50% (ref.)	4.21 (1684)	-	-
		50-70%	4.44 (709)	1.03 [0.94-1.13]	0.47
		70-90%	4.56 (728)	1.05 [0.95-1.16]	0.38
		90-100%	4.86 (388)	1.02 [0.90-1.17]	0.72
Model 3 + diabetes	Night	0-50% (ref.)	4.11 (1644)	-	-
N = 80058		50-70%	4.4 (705)	1.10 [1.00-1.20]*	0.04
		70-90%	4.73 (758)	1.16 [1.06-1.26]*	0.001
		90-100%	5.17 (414)	1.21 [1.08-1.35]*	<0.001
	Day	0-50% (ref.)	4.23 (1692)	-	-
		50-70%	4.43 (710)	1.03 [0.94-1.13]	0.48
		70-90%	4.57 (731)	1.05 [0.95-1.16]	0.31
		90-100%	4.85 (388)	1.04 [0.91-1.18]	0.60
Model 3 + hypertension	Night	0-50% (ref.)	4.11 (1644)	-	-
N = 80058		50-70%	4.4 (705)	1.10 [1.00-1.20]*	0.04
		70-90%	4.73 (758)	1.17 [1.07-1.27]*	<0.001
		90-100%	5.17 (414)	1.20 [1.08-1.34]*	0.001
	Day	0-50% (ref.)	4.23 (1692)	-	-
		50-70%	4.43 (710)	1.04 [0.95-1.14]	0.40
		70-90%	4.57 (731)	1.05 [0.95-1.16]	0.34
		90-100%	4.85 (388)	1.03 [0.91-1.18]	0.64
Model 3 + cholesterol ratio	Night	0-50% (ref.)	4.1 (1422)	-	-
N = 69288		50-70%	4.5 (624)	1.12 [1.02-1.23]*	0.02
		70-90%	4.68 (648)	1.16 [1.05-1.27]*	0.002
		90-100%	5.15 (357)	1.20 [1.07-1.36]*	0.002
	Day	0-50% (ref.)	4.16 (1442)	-	-
		50-70%	4.52 (627)	1.08 [0.98-1.19]	0.12
		70-90%	4.65 (644)	1.09 [0.98-1.21]	0.11
		90-100%	4.88 (338)	1.05 [0.91-1.21]	0.50
Model 3 + short sleep duration	Night	0-50% (ref.)	4.1 (1582)	-	-
N = 77077		50-70%	4.4 (679)	1.08 [0.99-1.18]	0.10

© 2025 Windred DP et al. *JAMA Network Open*.

		70-90%	4.75 (733)	1.13 [1.04-1.24]*	0.006
		90-100%	5.23 (403)	1.16 [1.04-1.30]*	0.01
	Day	0-50% (ref.)	4.21 (1621)	-	-
		50-70%	4.48 (690)	1.06 [0.96-1.16]	0.24
		70-90%	4.59 (707)	1.08 [0.97-1.19]	0.16
		90-100%	4.92 (379)	1.07 [0.93-1.22]	0.34
Model 3 + long sleep duration	Night	0-50% (ref.)	4.1 (1582)	-	-
N = 77077		50-70%	4.4 (679)	1.10 [1.01-1.21]*	0.036
		70-90%	4.75 (733)	1.18 [1.08-1.29]*	<0.001
		90-100%	5.23 (403)	1.25 [1.12-1.40]*	<0.001
	Day	0-50% (ref.)	4.21 (1621)	-	-
		50-70%	4.48 (690)	1.05 [0.95-1.15]	0.33
		70-90%	4.59 (707)	1.06 [0.96-1.17]	0.28
		90-100%	4.92 (379)	1.04 [0.91-1.19]	0.57
Model 3 + sleep efficiency	Night	0-50% (ref.)	4.1 (1582)	-	-
N = 77077		50-70%	4.4 (679)	1.10 [1.00-1.20]*	0.04
		70-90%	4.75 (733)	1.17 [1.07-1.28]*	<0.001
		90-100%	5.23 (403)	1.23 [1.10-1.38]*	<0.001
	Day	0-50% (ref.)	4.21 (1621)	-	-
		50-70%	4.48 (690)	1.05 [0.95-1.15]	0.32
		70-90%	4.59 (707)	1.06 [0.96-1.17]	0.26
		90-100%	4.92 (379)	1.04 [0.91-1.19]	0.55
Model 3, excluding shift workers	Night	0-50% (ref.)	4.2 (1543)	-	-
N = 73559		50-70%	4.46 (656)	1.12 [1.02-1.23]*	0.02
		70-90%	4.61 (678)	1.16 [1.05-1.27]*	0.002
		90-100%	5.15 (379)	1.25 [1.11-1.40]*	<0.001
	Day	0-50% (ref.)	4.23 (1557)	-	-
		50-70%	4.49 (660)	1.05 [0.95-1.15]	0.36
		70-90%	4.62 (680)	1.05 [0.95-1.16]	0.37
		90-100%	4.88 (359)	1.01 [0.88-1.15]	0.91
Model 3 - physical activity	Night	0-50% (ref.)	4.11 (1646)	-	-
N = 80186		50-70%	4.41 (708)	1.10 [1.01-1.21]*	0.03
		70-90%	4.74 (760)	1.18 [1.08-1.29]*	<0.001
		90-100%	5.16 (414)	1.25 [1.12-1.39]*	<0.001
	Day	0-50% (ref.)	4.23 (1694)	-	-
		50-70%	4.44 (712)	1.01 [0.92-1.11]	0.84

© 2025 Windred DP et al. *JAMA Network Open*.

	70-90%	4.56 (732)	0.99 [0.90-1.10]	0.89
	90-100%	4.86 (390)	0.93 [0.82-1.06]	0.27

Data are proportional hazards (95% CI). Model 3 was adjusted for: age, sex, ethnicity, photoperiod, yearly household income, area-level material deprivation, employment status, education, smoking status, alcohol consumption, healthy diet, physical activity, and urbanicity. * p<.05.

© 2025 Windred DP et al. *JAMA Network Open*.

eTable 7. Relationships of day and night light with myocardial infarction, adjusted for pre-existing cardiometabolic health, sleep, and excluding shift workers

		Percentile	Cases % (N)	HR [95% CI]	p-value
Model 3 + BMI	Night	0-50% (ref.)	1.73 (709)	-	-
N = 81982		50-70%	2.04 (335)	1.19 [1.04-1.36]*	0.009
		70-90%	2.15 (352)	1.24 [1.09-1.41]*	0.001
		90-100%	2.51 (206)	1.38 [1.18-1.62]*	<0.001
	Day	0-50% (ref.)	1.89 (776)	-	-
		50-70%	1.9 (312)	0.98 [0.85-1.12]	0.72
		70-90%	1.95 (320)	0.97 [0.83-1.13]	0.68
		90-100%	2.37 (194)	1.06 [0.88-1.28]	0.53
Model 3 + diabetes	Night	0-50% (ref.)	1.73 (710)	-	-
N = 82139		50-70%	2.05 (336)	1.19 [1.05-1.36]*	0.008
		70-90%	2.15 (354)	1.25 [1.09-1.42]*	<0.001
		90-100%	2.51 (206)	1.39 [1.18-1.63]*	<0.001
	Day	0-50% (ref.)	1.9 (779)	-	-
		50-70%	1.91 (313)	0.97 [0.85-1.12]	0.71
		70-90%	1.95 (320)	0.97 [0.84-1.13]	0.70
		90-100%	2.36 (194)	1.07 [0.89-1.29]	0.47
Model 3 + hypertension	Night	0-50% (ref.)	1.73 (710)	-	-
N = 82139		50-70%	2.05 (336)	1.20 [1.05-1.36]*	0.007
		70-90%	2.15 (354)	1.25 [1.10-1.43]*	<0.001
		90-100%	2.51 (206)	1.38 [1.18-1.62]*	<0.001
	Day	0-50% (ref.)	1.9 (779)	-	-
		50-70%	1.91 (313)	0.98 [0.85-1.12]	0.78
		70-90%	1.95 (320)	0.97 [0.83-1.12]	0.66
		90-100%	2.36 (194)	1.07 [0.88-1.29]	0.50
Model 3 + cholesterol ratio	Night	0-50% (ref.)	1.7 (603)	-	-
N = 71078		50-70%	2.05 (291)	1.21 [1.05-1.39]*	0.008
		70-90%	2.12 (301)	1.26 [1.09-1.45]*	0.002
		90-100%	2.45 (174)	1.37 [1.15-1.63]*	<0.001
	Day	0-50% (ref.)	1.84 (655)	-	-
		50-70%	1.9 (270)	1.01 [0.87-1.17]	0.93
		70-90%	1.93 (275)	0.99 [0.84-1.16]	0.91
		90-100%	2.38 (169)	1.10 [0.90-1.34]	0.37
Model 3 + short sleep duration	Night	0-50% (ref.)	1.73 (683)	-	-
N = 79079		50-70%	2.05 (325)	1.19 [1.04-1.35]*	0.02

© 2025 Windred DP et al. *JAMA Network Open*.

		70-90%	2.18 (345)	1.24 [1.09-1.42]*	0.001
		90-100%	2.52 (199)	1.35 [1.15-1.60]*	<0.001
	Day	0-50% (ref.)	1.91 (755)	-	-
		50-70%	1.91 (302)	0.97 [0.85-1.12]	0.69
		70-90%	1.93 (305)	0.96 [0.82-1.11]	0.56
		90-100%	2.4 (190)	1.08 [0.89-1.30]	0.45
Model 3 + long sleep duration	Night	0-50% (ref.)	1.73 (683)	-	-
N = 79079		50-70%	2.05 (325)	1.20 [1.05-1.37]*	0.007
		70-90%	2.18 (345)	1.27 [1.12-1.45]*	<0.001
		90-100%	2.52 (199)	1.42 [1.21-1.67]*	<0.001
	Day	0-50% (ref.)	1.91 (755)	-	-
		50-70%	1.91 (302)	0.97 [0.84-1.11]	0.62
		70-90%	1.93 (305)	0.94 [0.81-1.10]	0.45
		90-100%	2.4 (190)	1.06 [0.87-1.28]	0.58
Model 3 + sleep efficiency	Night	0-50% (ref.)	1.73 (683)	-	-
N = 79079		50-70%	2.05 (325)	1.20 [1.05-1.37]*	0.007
		70-90%	2.18 (345)	1.27 [1.11-1.45]*	<0.001
		90-100%	2.52 (199)	1.40 [1.19-1.65]*	<0.001
	Day	0-50% (ref.)	1.91 (755)	-	-
		50-70%	1.91 (302)	0.97 [0.84-1.11]	0.62
		70-90%	1.93 (305)	0.95 [0.81-1.10]	0.47
		90-100%	2.4 (190)	1.06 [0.87-1.28]	0.56
Model 3, excluding shift workers	Night	0-50% (ref.)	1.75 (661)	-	-
N = 75525		50-70%	2.07 (312)	1.23 [1.07-1.40]*	0.003
		70-90%	2.06 (311)	1.23 [1.07-1.41]*	0.003
		90-100%	2.52 (190)	1.45 [1.23-1.72]*	<0.001
	Day	0-50% (ref.)	1.89 (714)	-	-
		50-70%	1.89 (286)	0.98 [0.84-1.13]	0.73
		70-90%	1.93 (292)	0.96 [0.82-1.12]	0.60
		90-100%	2.41 (182)	1.07 [0.88-1.30]	0.51
Model 3 - physical activity	Night	0-50% (ref.)	1.73 (710)	-	-
N = 82268		50-70%	2.05 (338)	1.20 [1.06-1.37]*	0.005
		70-90%	2.16 (356)	1.27 [1.12-1.45]*	<0.001
		90-100%	2.5 (206)	1.44 [1.23-1.68]*	<0.001
	Day	0-50% (ref.)	1.9 (780)	-	-
		50-70%	1.9 (313)	0.95 [0.83-1.09]	0.43

© 2025 Windred DP et al. *JAMA Network Open*.

70-90%	1.95 (321)	0.91 [0.79-1.06]	0.23
90-100%	2.38 (196)	0.96 [0.80-1.16]	0.66

Data are proportional hazards (95% CI). Model 3 was adjusted for: age, sex, ethnicity, photoperiod, yearly household income, area-level material deprivation, employment status, education, smoking status, alcohol consumption, healthy diet, physical activity, and urbanicity. * p<.05.

© 2025 Windred DP et al. *JAMA Network Open*.

eTable 8. Relationships of day and night light with heart failure, adjusted for pre-existing cardiometabolic health, sleep, and excluding shift workers

		Percentile	Cases % (N)	HR [95% CI]	p-value
Model 3 + BMI	Night	0-50% (ref.)	1.83 (760)	-	-
N = 83245		50-70%	1.95 (325)	1.13 [0.99-1.28]	0.07
		70-90%	2.03 (338)	1.15 [1.01-1.31]*	0.04
		90-100%	2.59 (216)	1.36 [1.16-1.58]*	<0.001
	Day	0-50% (ref.)	1.96 (814)	-	-
		50-70%	2.04 (339)	1.00 [0.87-1.14]	0.97
		70-90%	1.83 (305)	0.87 [0.75-1.01]	0.07
		90-100%	2.17 (181)	0.93 [0.77-1.13]	0.46
Model 3 + diabetes	Night	0-50% (ref.)	1.83 (763)	-	-
N = 83402		50-70%	1.96 (327)	1.14 [1.00-1.30]	0.05
		70-90%	2.03 (339)	1.16 [1.02-1.33]*	0.02
		90-100%	2.61 (218)	1.41 [1.21-1.64]*	<0.001
	Day	0-50% (ref.)	1.96 (819)	-	-
		50-70%	2.03 (339)	0.99 [0.87-1.13]	0.90
		70-90%	1.85 (308)	0.88 [0.76-1.03]	0.11
		90-100%	2.17 (181)	0.95 [0.78-1.14]	0.56
Model 3 + hypertension	Night	0-50% (ref.)	1.83 (763)	-	-
N = 83402		50-70%	1.96 (327)	1.14 [1.00-1.30]*	0.04
		70-90%	2.03 (339)	1.17 [1.03-1.33]*	0.02
		90-100%	2.61 (218)	1.40 [1.20-1.63]*	<0.001
	Day	0-50% (ref.)	1.96 (819)	-	-
		50-70%	2.03 (339)	1.00 [0.88-1.14]	1.00
		70-90%	1.85 (308)	0.88 [0.76-1.02]	0.10
		90-100%	2.17 (181)	0.95 [0.78-1.14]	0.56
Model 3 + cholesterol ratio	Night	0-50% (ref.)	1.81 (653)	-	-
N = 72171		50-70%	2.02 (292)	1.19 [1.03-1.36]*	0.02
		70-90%	1.97 (284)	1.16 [1.01-1.34]*	0.04
		90-100%	2.54 (183)	1.41 [1.19-1.66]*	<0.001
	Day	0-50% (ref.)	1.94 (699)	-	-
		50-70%	1.97 (285)	0.98 [0.85-1.14]	0.83
		70-90%	1.88 (271)	0.90 [0.77-1.06]	0.21
		90-100%	2.18 (157)	0.94 [0.76-1.15]	0.52
Model 3 + short sleep duration	Night	0-50% (ref.)	1.83 (735)	-	-
N = 80280		50-70%	1.95 (313)	1.11 [0.97-1.27]	0.13

© 2025 Windred DP et al. *JAMA Network Open*.

		70-90%	2.03 (326)	1.13 [0.98-1.29]	0.08
		90-100%	2.65 (213)	1.34 [1.14-1.57]*	<0.001
	Day	0-50% (ref.)	1.95 (783)	-	-
		50-70%	2.06 (330)	1.03 [0.90-1.17]	0.72
		70-90%	1.85 (297)	0.91 [0.78-1.06]	0.22
		90-100%	2.2 (177)	0.99 [0.82-1.20]	0.92
Model 3 + long sleep duration	Night	0-50% (ref.)	1.83 (735)	-	-
N = 80280		50-70%	1.95 (313)	1.13 [0.99-1.30]	0.06
		70-90%	2.03 (326)	1.18 [1.03-1.34]*	0.02
		90-100%	2.65 (213)	1.46 [1.24-1.70]*	<0.001
	Day	0-50% (ref.)	1.95 (783)	-	-
		50-70%	2.06 (330)	1.01 [0.89-1.16]	0.84
		70-90%	1.85 (297)	0.89 [0.77-1.04]	0.14
		90-100%	2.2 (177)	0.96 [0.79-1.16]	0.68
Model 3 + sleep efficiency	Night	0-50% (ref.)	1.83 (735)	-	-
N = 80280		50-70%	1.95 (313)	1.13 [0.99-1.29]	0.07
		70-90%	2.03 (326)	1.17 [1.02-1.33]*	0.02
		90-100%	2.65 (213)	1.43 [1.22-1.67]*	<0.001
	Day	0-50% (ref.)	1.95 (783)	-	-
		50-70%	2.06 (330)	1.02 [0.89-1.16]	0.82
		70-90%	1.85 (297)	0.90 [0.77-1.04]	0.16
		90-100%	2.2 (177)	0.97 [0.80-1.17]	0.73
Model 3, excluding shift workers	Night	0-50% (ref.)	1.9 (728)	-	-
N = 76716		50-70%	2.03 (311)	1.17 [1.02-1.34]*	0.02
		70-90%	2.03 (311)	1.17 [1.02-1.34]*	0.02
		90-100%	2.54 (195)	1.40 [1.19-1.65]*	<0.001
	Day	0-50% (ref.)	2.01 (770)	-	-
		50-70%	2.07 (317)	0.99 [0.86-1.14]	0.90
		70-90%	1.87 (287)	0.87 [0.74-1.01]	0.07
		90-100%	2.23 (171)	0.92 [0.76-1.12]	0.40
Model 3 - physical activity	Night	0-50% (ref.)	1.83 (764)	-	-
N = 83532		50-70%	1.98 (331)	1.16 [1.02-1.32]*	0.03
		70-90%	2.03 (339)	1.19 [1.05-1.36]*	0.008
		90-100%	2.61 (218)	1.49 [1.28-1.74]*	<0.001
	Day	0-50% (ref.)	1.97 (821)	-	-
		50-70%	2.04 (341)	0.95 [0.84-1.09]	0.49

© 2025 Windred DP et al. *JAMA Network Open*.

	70-90%	1.85 (309)	0.80 [0.69-0.93]*	0.003
	90-100%	2.17 (181)	0.78 [0.65-0.95]*	0.01

Data are proportional hazards (95% CI). Model 3 was adjusted for: age, sex, ethnicity, photoperiod, yearly household income, area-level material deprivation, employment status, education, smoking status, alcohol consumption, healthy diet, physical activity, and urbanicity. * p<.05.

© 2025 Windred DP et al. *JAMA Network Open*.

eTable 9. Relationships of day and night light with atrial fibrillation, adjusted for pre-existing cardiometabolic health, sleep, and excluding shift workers

		Percentile	Cases % (N)	HR [95% CI]	p-value
Model 3 + BMI	Night	0-50% (ref.)	4.17 (1696)	-	-
N = 81382		50-70%	4.21 (686)	1.06 [0.97-1.16]	0.20
		70-90%	4.29 (699)	1.06 [0.97-1.16]	0.18
		90-100%	5.16 (420)	1.22 [1.09-1.36]*	<0.001
	Day	0-50% (ref.)	4.1 (1668)	-	-
		50-70%	4.42 (720)	1.04 [0.95-1.14]	0.38
		70-90%	4.34 (707)	0.99 [0.89-1.09]	0.83
		90-100%	4.99 (406)	1.01 [0.89-1.15]	0.83
Model 3 + diabetes	Night	0-50% (ref.)	4.17 (1700)	-	-
N = 81537		50-70%	4.24 (692)	1.08 [0.99-1.18]	0.10
		70-90%	4.3 (701)	1.08 [0.99-1.18]	0.08
		90-100%	5.18 (422)	1.27 [1.13-1.41]*	<0.001
	Day	0-50% (ref.)	4.12 (1678)	-	-
		50-70%	4.43 (722)	1.04 [0.95-1.14]	0.35
		70-90%	4.34 (707)	0.99 [0.90-1.10]	0.89
		90-100%	5 (408)	1.03 [0.90-1.17]	0.68
Model 3 + hypertension	Night	0-50% (ref.)	4.17 (1700)	-	-
N = 81537		50-70%	4.24 (692)	1.08 [0.99-1.18]	0.09
		70-90%	4.3 (701)	1.09 [0.99-1.19]	0.07
		90-100%	5.18 (422)	1.26 [1.13-1.40]*	<0.001
	Day	0-50% (ref.)	4.12 (1678)	-	-
		50-70%	4.43 (722)	1.05 [0.96-1.15]	0.30
		70-90%	4.34 (707)	0.99 [0.90-1.10]	0.87
		90-100%	5 (408)	1.03 [0.90-1.17]	0.66
Model 3 + cholesterol ratio	Night	0-50% (ref.)	4.16 (1466)	-	-
N = 70548		50-70%	4.35 (614)	1.11 [1.01-1.22]*	0.04
		70-90%	4.17 (588)	1.06 [0.96-1.17]	0.23
		90-100%	5.12 (361)	1.26 [1.12-1.42]*	<0.001
	Day	0-50% (ref.)	4.08 (1440)	-	-
		50-70%	4.42 (624)	1.06 [0.97-1.17]	0.21
		70-90%	4.32 (609)	1.00 [0.90-1.12]	0.94
		90-100%	5.05 (356)	1.05 [0.91-1.20]	0.51
Model 3 + short sleep duration	Night	0-50% (ref.)	4.17 (1637)	-	-
N = 78488		50-70%	4.27 (671)	1.08 [0.98-1.18]	0.12

© 2025 Windred DP et al. *JAMA Network Open*.

		70-90%	4.33 (679)	1.08 [0.98-1.18]	0.11
		90-100%	5.22 (410)	1.25 [1.11-1.40]*	<0.001
	Day	0-50% (ref.)	4.12 (1616)	-	-
		50-70%	4.46 (700)	1.05 [0.96-1.16]	0.27
		70-90%	4.34 (681)	1.00 [0.90-1.10]	0.95
		90-100%	5.1 (400)	1.05 [0.92-1.19]	0.50
Model 3 + long sleep duration	Night	0-50% (ref.)	4.17 (1637)	-	-
N = 78488		50-70%	4.27 (671)	1.09 [0.99-1.19]	0.07
		70-90%	4.33 (679)	1.10 [1.00-1.20]*	0.04
		90-100%	5.22 (410)	1.30 [1.16-1.45]*	<0.001
	Day	0-50% (ref.)	4.12 (1616)	-	-
		50-70%	4.46 (700)	1.05 [0.96-1.15]	0.31
		70-90%	4.34 (681)	0.99 [0.89-1.09]	0.82
		90-100%	5.1 (400)	1.03 [0.91-1.18]	0.64
Model 3 + sleep efficiency	Night	0-50% (ref.)	4.17 (1637)	-	-
N = 78488		50-70%	4.27 (671)	1.09 [0.99-1.19]	0.08
		70-90%	4.33 (679)	1.09 [1.00-1.20]	0.06
		90-100%	5.22 (410)	1.28 [1.15-1.43]*	<0.001
	Day	0-50% (ref.)	4.12 (1616)	-	-
		50-70%	4.46 (700)	1.05 [0.96-1.15]	0.31
		70-90%	4.34 (681)	0.99 [0.89-1.10]	0.84
		90-100%	5.1 (400)	1.04 [0.91-1.18]	0.61
Model 3, excluding shift workers	Night	0-50% (ref.)	4.31 (1616)	-	-
N = 74943		50-70%	4.39 (658)	1.10 [1.01-1.21]*	0.03
		70-90%	4.38 (656)	1.10 [1.01-1.21]*	0.04
		90-100%	5.11 (383)	1.26 [1.13-1.41]*	<0.001
	Day	0-50% (ref.)	4.24 (1587)	-	-
		50-70%	4.47 (670)	1.03 [0.94-1.13]	0.57
		70-90%	4.48 (672)	0.99 [0.89-1.10]	0.85
		90-100%	5.12 (384)	1.00 [0.88-1.15]	0.97
Model 3 - physical activity	Night	0-50% (ref.)	4.17 (1701)	-	-
N = 81666		50-70%	4.25 (694)	1.09 [0.99-1.19]	0.07
		70-90%	4.3 (703)	1.10 [1.01-1.20]*	0.04
		90-100%	5.17 (422)	1.30 [1.16-1.45]*	<0.001
	Day	0-50% (ref.)	4.11 (1679)	-	-
		50-70%	4.43 (723)	1.03 [0.94-1.12]	0.58

© 2025 Windred DP et al. *JAMA Network Open*.

	70-90%	4.33 (708)	0.95 [0.86-1.05]	0.35
	90-100%	5.02 (410)	0.95 [0.84-1.08]	0.47

Data are proportional hazards (95% CI). Model 3 was adjusted for: age, sex, ethnicity, photoperiod, yearly household income, area-level material deprivation, employment status, education, smoking status, alcohol consumption, healthy diet, physical activity, and urbanicity. * p<.05.

© 2025 Windred DP et al. *JAMA Network Open*.

eTable 10. Relationships of day and night light with stroke, adjusted for pre-existing cardiometabolic health, sleep, and excluding shift workers

		Percentile	Cases % (N)	HR [95% CI]	p-value
Model 3 + BMI	Night	0-50% (ref.)	1.33 (549)	-	-
N = 82773		50-70%	1.33 (220)	1.08 [0.93-1.27]	0.32
		70-90%	1.33 (221)	1.08 [0.92-1.27]	0.33
		90-100%	1.66 (137)	1.28 [1.06-1.56]*	0.01
	Day	0-50% (ref.)	1.39 (574)	-	-
		50-70%	1.29 (213)	0.90 [0.77-1.07]	0.23
		70-90%	1.41 (234)	0.98 [0.83-1.17]	0.86
		90-100%	1.28 (106)	0.81 [0.64-1.03]	0.09
Model 3 + diabetes	Night	0-50% (ref.)	1.33 (550)	-	-
N = 82931		50-70%	1.34 (222)	1.09 [0.93-1.27]	0.30
		70-90%	1.34 (222)	1.08 [0.92-1.26]	0.37
		90-100%	1.65 (137)	1.27 [1.05-1.54]*	0.01
	Day	0-50% (ref.)	1.39 (575)	-	-
		50-70%	1.29 (214)	0.91 [0.77-1.07]	0.25
		70-90%	1.42 (235)	0.99 [0.83-1.18]	0.94
		90-100%	1.29 (107)	0.83 [0.65-1.05]	0.12
Model 3 + hypertension	Night	0-50% (ref.)	1.33 (550)	-	-
N = 82931		50-70%	1.34 (222)	1.09 [0.93-1.27]	0.29
		70-90%	1.34 (222)	1.08 [0.92-1.26]	0.36
		90-100%	1.65 (137)	1.26 [1.04-1.53]*	0.02
	Day	0-50% (ref.)	1.39 (575)	-	-
		50-70%	1.29 (214)	0.91 [0.77-1.07]	0.27
		70-90%	1.42 (235)	0.99 [0.83-1.18]	0.93
		90-100%	1.29 (107)	0.83 [0.65-1.05]	0.12
Model 3 + cholesterol ratio	Night	0-50% (ref.)	1.38 (497)	-	-
N = 71769		50-70%	1.38 (198)	1.06 [0.9-1.25]	0.47
		70-90%	1.36 (195)	1.05 [0.89-1.24]	0.55
		90-100%	1.62 (116)	1.18 [0.96-1.46]	0.11
	Day	0-50% (ref.)	1.43 (513)	-	-
		50-70%	1.32 (189)	0.90 [0.76-1.07]	0.23
		70-90%	1.44 (206)	0.97 [0.81-1.17]	0.75
		90-100%	1.37 (98)	0.84 [0.65-1.08]	0.17
Model 3 + short sleep duration	Night	0-50% (ref.)	1.32 (528)	-	-
N = 79829		50-70%	1.33 (212)	1.06 [0.9-1.24]	0.48

© 2025 Windred DP et al. *JAMA Network Open*.

		70-90%	1.37 (218)	1.07 [0.91-1.26]	0.42
		90-100%	1.64 (131)	1.20 [0.98-1.47]	0.07
	Day	0-50% (ref.)	1.38 (550)	-	-
		50-70%	1.31 (209)	0.93 [0.78-1.09]	0.36
		70-90%	1.43 (228)	1.01 [0.84-1.2]	0.95
		90-100%	1.28 (102)	0.82 [0.64-1.05]	0.12
Model 3 + long sleep duration	Night	0-50% (ref.)	1.32 (528)	-	-
N = 79829		50-70%	1.33 (212)	1.08 [0.92-1.27]	0.34
		70-90%	1.37 (218)	1.12 [0.96-1.32]	0.16
		90-100%	1.64 (131)	1.30 [1.07-1.58]*	0.009
	Day	0-50% (ref.)	1.38 (550)	-	-
		50-70%	1.31 (209)	0.92 [0.78-1.09]	0.32
		70-90%	1.43 (228)	0.99 [0.83-1.18]	0.91
		90-100%	1.28 (102)	0.80 [0.62-1.02]	0.07
Model 3 + sleep efficiency	Night	0-50% (ref.)	1.32 (528)	-	-
N = 79829		50-70%	1.33 (212)	1.08 [0.92-1.27]	0.35
		70-90%	1.37 (218)	1.11 [0.94-1.3]	0.21
		90-100%	1.64 (131)	1.28 [1.05-1.56]*	0.01
	Day	0-50% (ref.)	1.38 (550)	-	-
		50-70%	1.31 (209)	0.92 [0.78-1.08]	0.32
		70-90%	1.43 (228)	0.99 [0.83-1.18]	0.92
		90-100%	1.28 (102)	0.80 [0.63-1.03]	0.08
Model 3, excluding shift workers	Night	0-50% (ref.)	1.38 (525)	-	-
N = 76261		50-70%	1.37 (209)	1.09 [0.93-1.29]	0.27
		70-90%	1.39 (212)	1.11 [0.95-1.31]	0.19
		90-100%	1.6 (122)	1.24 [1.01-1.52]*	0.04
	Day	0-50% (ref.)	1.43 (545)	-	-
		50-70%	1.32 (201)	0.89 [0.76-1.06]	0.20
		70-90%	1.46 (222)	0.97 [0.81-1.16]	0.72
		90-100%	1.31 (100)	0.78 [0.61-1]	0.05
Model 3 - physical activity	Night	0-50% (ref.)	1.33 (551)	-	-
N = 83062		50-70%	1.35 (224)	1.10 [0.94-1.28]	0.25
		70-90%	1.34 (222)	1.08 [0.93-1.27]	0.31
		90-100%	1.65 (137)	1.30 [1.07-1.57]*	0.007
	Day	0-50% (ref.)	1.39 (577)	-	-
		50-70%	1.29 (215)	0.89 [0.75-1.05]	0.16

© 2025 Windred DP et al. *JAMA Network Open*.

70-90%	1.41 (235)	0.94 [0.79-1.12]	0.48
90-100%	1.29 (107)	0.75 [0.59-0.95]*	0.02

Data are proportional hazards (95% CI). Model 3 was adjusted for: age, sex, ethnicity, photoperiod, yearly household income, area-level material deprivation, employment status, education, smoking status, alcohol consumption, healthy diet, physical activity, and urbanicity. * p<.05.

© 2025 Windred DP et al. *JAMA Network Open*.

eTable 11. Interaction of chronotype and night light exposure for cardiovascular risks

		Coronary artery disease		Myocardial infarction		Heart failure		Atrial fibrillation		Stroke	
		**HR [95% CI]**	**p-value**	**HR [95% CI]**	**p-value**	**HR [95% CI]**	**p-value**	**HR [95% CI]**	**p-value**	**HR [95% CI]**	**p-value**
**Model 3 + Chronotype**	Night light	1.06 [1.03-1.08]*	<0.001	1.08 [1.04-1.11]*	<0.001	1.08 [1.05-1.12]*	<0.001	1.05 [1.02-1.07]*	<0.001	1.05 [1.01-1.09]*	0.02
	Evening chronotype	1.00 [0.93-1.07]	0.92	0.94 [0.84-1.05]	0.29	0.94 [0.85-1.05]	0.28	1.00 [0.93-1.07]	0.94	0.99 [0.87-1.13]	0.85
**Model 3 + Chronotype*Night light**	Night light	1.07 [1.04-1.11]*	<0.001	1.09 [1.04-1.14]*	<0.001	1.07 [1.03-1.12]*	0.002	1.03 [1.00-1.07]*	0.04	1.06 [1.00-1.11]*	0.05
	Chronotype	1.06 [0.95-1.18]	0.30	0.99 [0.84-1.17]	0.89	0.90 [0.77-1.06]	0.22	0.94 [0.84-1.05]	0.25	1.02 [0.84-1.23]	0.88
	Night light*Evening chronotype	0.97 [0.92-1.01]	0.14	0.97 [0.91-1.04]	0.46	1.02 [0.96-1.09]	0.49	1.04 [0.99-1.08]	0.14	0.98 [0.91-1.07]	0.70

*p<.05. Hazard ratios [95% CIs] are adjusted for Model 3 covariates (age, sex, ethnicity, photoperiod, education, employment, income, deprivation, physical activity, smoking status, alcohol consumption, diet, and urbanicity). Hazard ratios for chronotype are for evening types compared to morning types, and hazard ratios for night light are per one unit increase in log-transformed light exposure.

© 2025 Windred DP et al. *JAMA Network Open*.

eTable 12. Risk of cardiovascular outcomes across Models 1-3 after excluding participants with any cardiovascular disease prior to light tracking

			**Coronary artery disease**		**Myocardial infarction**		**Heart failure**		**Atrial fibrillation**		**Stroke**	
		Percentile	HR [95% CI]	p-value	HR [95% CI]	p-value	HR [95% CI]	p-value	HR [95% CI]	p-value	HR [95% CI]	p-value
Model 1	Night	0-50% (ref.)	-	-	-	-	-	-	-	-	-	-
		50-70%	1.10 [1.01-1.21]*	0.04	1.12 [0.97-1.30]	0.11	1.16 [0.99-1.35]	0.06	1.11 [1.01-1.21]*	0.03	1.16 [0.98-1.37]	0.09
		70-90%	1.18 [1.08-1.29]*	<0.001	1.25 [1.08-1.44]*	0.002	1.16 [0.99-1.35]	0.07	1.09 [1.00-1.20]	0.06	1.14 [0.96-1.35]	0.14
		90-100%	1.35 [1.21-1.51]*	<0.001	1.51 [1.27-1.79]*	<0.001	1.57 [1.31-1.88]*	<0.001	1.29 [1.15-1.45]*	<0.001	1.29 [1.05-1.59]*	0.02
	Day	0-50% (ref.)	-	-	-	-	-	-	-	-	-	-
		50-70%	0.98 [0.89-1.08]	0.65	0.95 [0.82-1.10]	0.48	0.98 [0.84-1.15]	0.83	1.01 [0.92-1.11]	0.85	0.94 [0.79-1.12]	0.49
		70-90%	0.96 [0.87-1.07]	0.48	0.92 [0.78-1.08]	0.31	0.76 [0.64-0.91]*	0.003	0.93 [0.84-1.03]	0.17	0.87 [0.72-1.05]	0.15
		90-100%	0.86 [0.75-0.98]*	0.02	0.90 [0.74-1.11]	0.32	0.75 [0.60-0.93]*	0.01	0.95 [0.83-1.09]	0.46	0.73 [0.56-0.94]*	0.02
Model 2	Night	0-50% (ref.)	-	-	-	-	-	-	-	-	-	-
		50-70%	1.10 [1.00-1.21]*	0.04	1.14 [0.98-1.32]	0.08	1.17 [1.00-1.36]	0.05	1.11 [1.01-1.22]*	0.02	1.16 [0.98-1.38]	0.08
		70-90%	1.18 [1.07-1.29]*	<0.001	1.26 [1.09-1.46]*	0.002	1.15 [0.98-1.35]	0.09	1.10 [1.00-1.21]*	0.05	1.16 [0.97-1.37]	0.10
		90-100%	1.32 [1.18-1.48]*	<0.001	1.49 [1.25-1.77]*	<0.001	1.56 [1.30-1.87]*	<0.001	1.29 [1.15-1.45]*	<0.001	1.31 [1.06-1.62]*	0.01
	Day	0-50% (ref.)	-	-	-	-	-	-	-	-	-	-
		50-70%	0.99 [0.90-1.08]	0.78	0.95 [0.82-1.11]	0.51	1.01 [0.86-1.18]	0.90	1.02 [0.92-1.12]	0.72	0.92 [0.78-1.10]	0.38
		70-90%	0.98 [0.88-1.08]	0.68	0.93 [0.79-1.09]	0.36	0.78 [0.65-0.94]*	0.008	0.94 [0.84-1.04]	0.23	0.89 [0.73-1.07]	0.21
		90-100%	0.88 [0.77-1.01]	0.07	0.92 [0.75-1.14]	0.45	0.78 [0.62-0.99]*	0.04	0.96 [0.84-1.10]	0.53	0.74 [0.57-0.96]*	0.02
Model 3	Night	0-50% (ref.)	-	-	-	-	-	-	-	-	-	-
		50-70%	1.09 [0.99-1.20]	0.08	1.15 [0.99-1.33]	0.08	1.17 [1.00-1.37]	0.05	1.10 [1.00-1.21]	0.06	1.15 [0.97-1.37]	0.10
		70-90%	1.17 [1.06-1.28]*	0.001	1.26 [1.09-1.46]*	0.002	1.14 [0.97-1.34]	0.12	1.07 [0.97-1.18]	0.16	1.14 [0.96-1.36]	0.14
		90-100%	1.26 [1.13-1.42]*	<0.001	1.47 [1.23-1.75]*	<0.001	1.49 [1.23-1.79]*	<0.001	1.27 [1.13-1.43]*	<0.001	1.30 [1.05-1.61]*	0.02
	Day	0-50% (ref.)	-	-	-	-	-	-	-	-	-	-
		50-70%	1.02 [0.93-1.13]	0.67	0.99 [0.85-1.16]	0.92	1.07 [0.91-1.25]	0.44	1.05 [0.95-1.15]	0.36	0.95 [0.80-1.14]	0.57
		70-90%	1.06 [0.95-1.17]	0.30	0.99 [0.84-1.17]	0.91	0.89 [0.74-1.06]	0.20	0.99 [0.89-1.10]	0.81	0.93 [0.76-1.13]	0.44
		90-100%	1.01 [0.88-1.17]	0.84	1.05 [0.84-1.30]	0.68	0.94 [0.74-1.19]	0.62	1.03 [0.90-1.19]	0.65	0.79 [0.60-1.03]	0.08

*p<.05. Hazard ratios [95% CIs] adjusted for age, sex, ethnicity, and photoperiod (Model 1); additionally adjusted for education, employment, income, and deprivation (Model 2); and further adjusted for physical activity, smoking status, alcohol consumption, diet, and urbanicity (Model 3).

© 2025 Windred DP et al. *JAMA Network Open*.

eTable 13. Risk of cardiovascular outcomes across Models 1-3 from UK Biobank enrolment (commencing March 2006) to study administrative endpoint (November 2022)

			Coronary artery disease		Myocardial infarction		Heart failure		Atrial fibrillation		Stroke	
		Percentile	HR [95% CI]	p-value	HR [95% CI]	p-value	HR [95% CI]	p-value	HR [95% CI]	p-value	HR [95% CI]	p-value
Model 1	Night	0-50% (ref.)	-	-	-	-	-	-	-	-	-	-
		50-70%	1.11 [1.03-1.19]*	0.004	1.20 [1.07-1.33]*	0.001	1.14 [1.02-1.28]*	0.02	1.09 [1.01-1.18]*	0.02	1.07 [0.93-1.23]	0.32
		70-90%	1.16 [1.08-1.24]*	<0.001	1.19 [1.07-1.33]*	0.002	1.21 [1.08-1.36]*	<0.001	1.13 [1.05-1.22]*	<0.001	1.09 [0.95-1.25]	0.23
		90-100%	1.29 [1.18-1.41]*	<0.001	1.42 [1.25-1.63]*	<0.001	1.53 [1.33-1.75]*	<0.001	1.35 [1.23-1.48]*	<0.001	1.29 [1.09-1.52]*	0.004
	Day	0-50% (ref.)	-	-	-	-	-	-	-	-	-	-
		50-70%	0.94 [0.87-1.01]	0.10	0.88 [0.79-0.99]*	0.04	0.89 [0.79-1.00]*	0.05	0.98 [0.91-1.06]	0.68	0.90 [0.78-1.04]	0.17
		70-90%	0.92 [0.85-1.00]*	0.04	0.88 [0.78-1.00]*	0.05	0.75 [0.66-0.86]*	<0.001	0.92 [0.85-1.00]	0.05	0.92 [0.79-1.08]	0.31
		90-100%	0.89 [0.80-0.98]*	0.02	0.92 [0.79-1.08]	0.32	0.73 [0.62-0.86]*	<0.001	0.92 [0.83-1.02]	0.13	0.72 [0.58-0.88]*	0.002
Model 2	Night	0-50% (ref.)	-	-	-	-	-	-	-	-	-	-
		50-70%	1.11 [1.03-1.19]*	0.006	1.20 [1.08-1.34]*	<0.001	1.15 [1.02-1.29]*	0.02	1.09 [1.01-1.18]*	0.02	1.08 [0.94-1.25]	0.26
		70-90%	1.15 [1.07-1.23]*	<0.001	1.19 [1.07-1.33]*	0.002	1.21 [1.08-1.35]*	0.001	1.14 [1.06-1.23]*	<0.001	1.10 [0.95-1.26]	0.20
		90-100%	1.27 [1.16-1.39]*	<0.001	1.40 [1.22-1.61]*	<0.001	1.50 [1.30-1.72]*	<0.001	1.35 [1.23-1.48]*	<0.001	1.30 [1.10-1.55]*	0.003
	Day	0-50% (ref.)	-	-	-	-	-	-	-	-	-	-
		50-70%	0.94 [0.87-1.02]	0.13	0.89 [0.79-1.00]*	0.05	0.91 [0.81-1.02]	0.12	0.99 [0.92-1.07]	0.82	0.90 [0.78-1.04]	0.14
		70-90%	0.93 [0.85-1.01]	0.07	0.89 [0.78-1.01]	0.07	0.78 [0.69-0.89]*	<0.001	0.93 [0.86-1.02]	0.11	0.95 [0.81-1.11]	0.51
		90-100%	0.90 [0.81-1.00]	0.06	0.94 [0.80-1.10]	0.45	0.77 [0.65-0.91]*	0.002	0.93 [0.83-1.04]	0.19	0.73 [0.59-0.90]*	0.004
Model 3	Night	0-50% (ref.)	-	-	-	-	-	-	-	-	-	-
		50-70%	1.09 [1.02-1.18]*	0.02	1.19 [1.06-1.33]*	0.002	1.14 [1.02-1.29]*	0.03	1.08 [1.00-1.17]*	0.05	1.09 [0.95-1.26]	0.21
		70-90%	1.13 [1.05-1.21]*	0.001	1.18 [1.05-1.32]*	0.005	1.19 [1.06-1.34]*	0.003	1.12 [1.04-1.21]*	0.004	1.08 [0.93-1.24]	0.31
		90-100%	1.21 [1.11-1.33]*	<0.001	1.35 [1.18-1.55]*	<0.001	1.41 [1.23-1.63]*	<0.001	1.32 [1.20-1.44]*	<0.001	1.28 [1.08-1.53]*	0.005
	Day	0-50% (ref.)	-	-	-	-	-	-	-	-	-	-
		50-70%	0.98 [0.91-1.06]	0.58	0.93 [0.82-1.05]	0.23	0.96 [0.85-1.09]	0.56	1.03 [0.95-1.11]	0.47	0.94 [0.81-1.09]	0.42
		70-90%	1.01 [0.93-1.10]	0.74	0.97 [0.86-1.11]	0.70	0.90 [0.79-1.03]	0.13	1.00 [0.91-1.09]	0.95	1.02 [0.87-1.20]	0.77
		90-100%	1.06 [0.95-1.18]	0.31	1.10 [0.93-1.29]	0.27	0.97 [0.81-1.15]	0.72	1.03 [0.92-1.15]	0.59	0.83 [0.67-1.04]	0.11

*p<.05. Hazard ratios [95% CIs] adjusted for age, sex, ethnicity, and photoperiod (Model 1); additionally adjusted for education, employment, income, and deprivation (Model 2); and further adjusted for physical activity, smoking status, alcohol consumption, diet, and urbanicity (Model 3)

© 2025 Windred DP et al. *JAMA Network Open*.

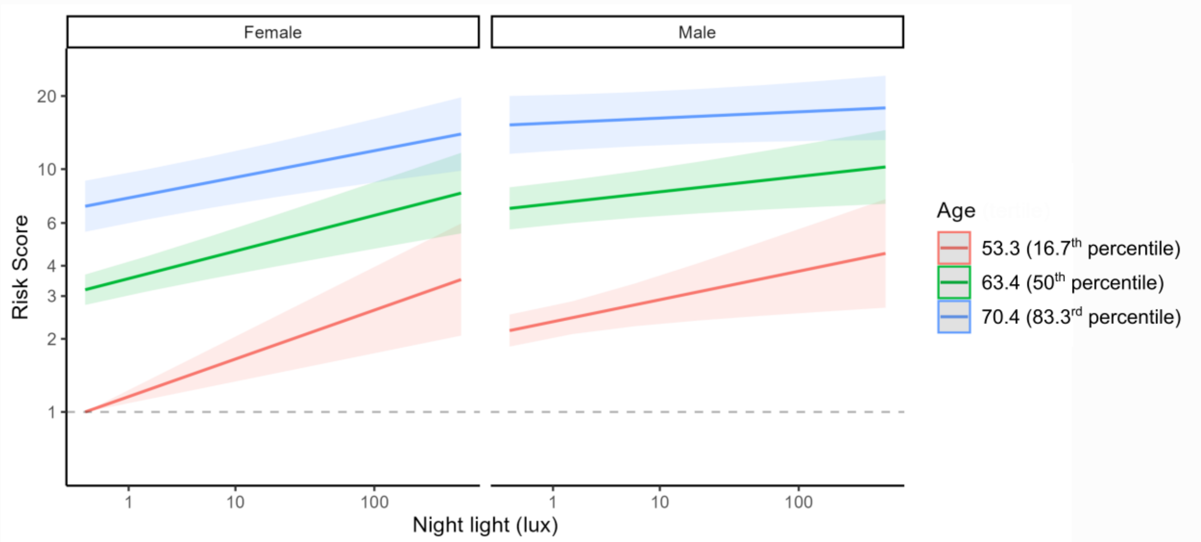

eFigure 1. Relationship of night light exposure with risk of heart failure, according to participant age and sex. This figure captures the marginal effects of light exposure on heart failure risk at specified ages, for males and females, and is derived from Table 3: Model 3 + Night light*Sex + Night light*Age.

© 2025 Windred DP et al. *JAMA Network Open*.

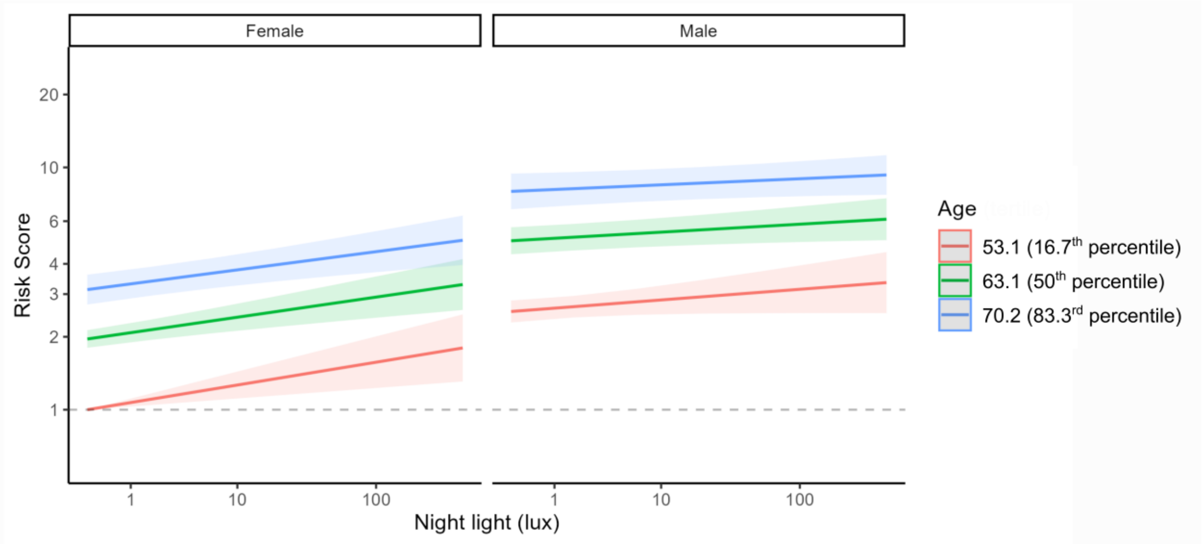

eFigure 2. Relationship of night light exposure with risk of coronary artery disease, according to participant age and sex. This figure captures the marginal effects of light exposure on coronary artery disease risk at specified ages, for males and females, and is derived from Table 3: Model 3 + Night light*Sex + Night light*Age.

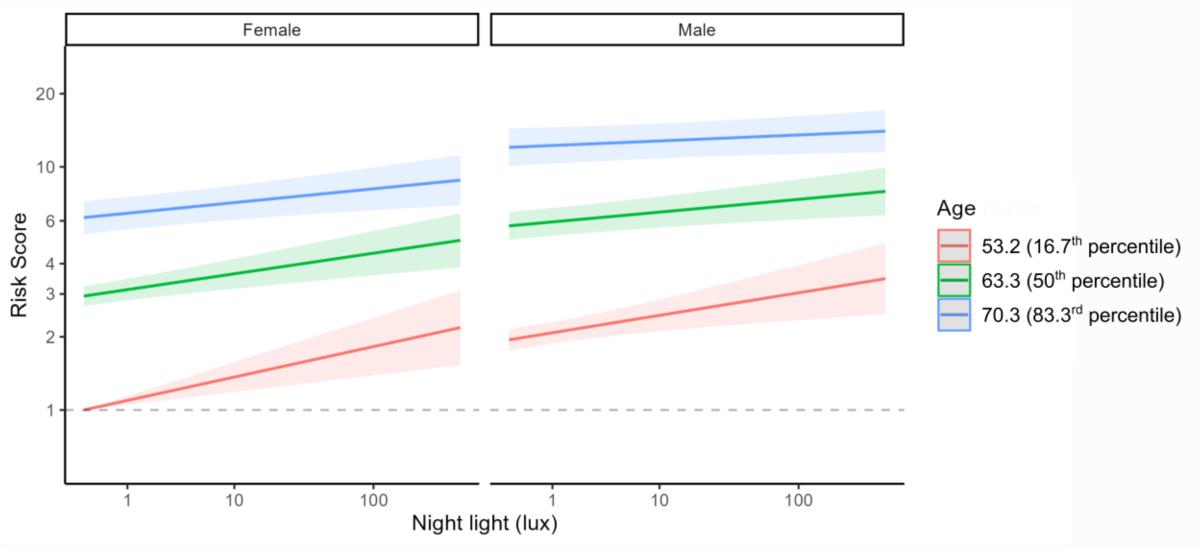

eFigure 3. Relationship of night light exposure with risk of atrial fibrillation, according to participant age and sex. This figure captures the marginal effects of light exposure on atrial fibrillation risk at specified ages, for males and females, and is derived from Table 3: Model 3 + Night light*Sex + Night light*Age

© 2025 Windred DP et al. *JAMA Network Open*.

eReferences

1. Burns AC, Windred DP, Rutter MK, et al. Day and night light exposure are associated with psychiatric disorders: an objective light study in> 85,000 people. *Nature Mental Health*. 2023;1(11):853-862.
2. Kurki MI, Karjalainen J, Palta P, et al. FinnGen provides genetic insights from a well-phenotyped isolated population. *Nature*. 2023;613(7944):508-518.
3. Levin MG, Tsao NL, Singhal P, et al. Genome-wide association and multi-trait analyses characterize the common genetic architecture of heart failure. *Nature Communications*. 2022;13(1):6914.
4. Mishra A, Malik R, Hachiya T, et al. Stroke genetics informs drug discovery and risk prediction across ancestries. *Nature*. 2022;611(7934):115-123.
5. Ge T, Chen C-Y, Ni Y, Feng Y-CA, Smoller JW. Polygenic prediction via Bayesian regression and continuous shrinkage priors. *Nature Communications*. 2019/04/16 2019;10(1):1776. doi:10.1038/s41467-019-09718-5
6. Chang CC, Chow CC, Tellier LC, Vattikuti S, Purcell SM, Lee JJ. Second-generation PLINK: rising to the challenge of larger and richer datasets. *Gigascience*. 2015;4(1):s13742-015-0047-8.
7. Koenig Z, Yohannes MT, Nkambule LL, et al. A harmonized public resource of deeply sequenced diverse human genomes. *Genome Research*. 2024;
8. Patterson N, Price AL, Reich D. Population structure and eigenanalysis. *PLoS genetics*. 2006;2(12):e190.
9. Purcell S, Neale B, Todd-Brown K, et al. PLINK: a tool set for whole-genome association and population-based linkage analyses. *The American journal of human genetics*. 2007;81(3):559-575.
10. Manichaikul A, Mychaleckyj JC, Rich SS, Daly K, Sale M, Chen W-M. Robust relationship inference in genome-wide association studies. *Bioinformatics*. 2010;26(22):2867-2873.
11. Said MA, Verweij N, van der Harst P. Associations of combined genetic and lifestyle risks with incident cardiovascular disease and diabetes in the UK Biobank Study. *JAMA cardiology*. 2018;3(8):693-702.
12. Cappuccio FP, Barbato A, Kerry SM. Hypertension, diabetes and cardiovascular risk in ethnic minorities in the UK. *The British Journal of Diabetes & Vascular Disease*. 2003;3(4):286-293.

© 2025 Windred DP et al. *JAMA Network Open*.
