# Supplementary material for: Light Exposure at Night and Cardiovascular Disease Incidence
Source: JAMA Netw Open. 2025 Oct 23;8(10):e2539031. doi: 10.1001/jamanetworkopen.2025.39031 (PMC12550636; doi:10.1001/jamanetworkopen.2025.39031)
Supplement: Supplement 2. — Data Sharing Statement [file jamanetwopen-e2539031-s002.pdf]

Data Sharing Statement

Windred. Light Exposure at Night and Cardiovascular Disease Incidence. *JAMA Netw Open*. Published October 23, 2025. doi:10.1001/jamanetworkopen.2025.39031

Data
Data available: Yes
Data types: Deidentified participant data
How to access data: Data can be accessed at the following link: https://biobank.ndph.ox.ac.uk/showcase/
When available: With publication

Supporting Documents
Document types: None

Additional Information
Who can access the data: Data will be made available to researchers, following an approved application.
Types of analyses: Data usage purposes will be specified in each researcher's UK Biobank application.
Mechanisms of data availability: Data are made available by the UK Biobank team upon application.
